# Computer-Aided Multi-Epitope Vaccine Design against *Enterobacter xiangfangensis*

**DOI:** 10.3390/ijerph19137723

**Published:** 2022-06-23

**Authors:** Abdulrahman Alshammari, Metab Alharbi, Abdullah Alghamdi, Saif Ali Alharbi, Usman Ali Ashfaq, Muhammad Tahir ul Qamar, Asad Ullah, Muhammad Irfan, Amjad Khan, Sajjad Ahmad

**Affiliations:** 1 Department of Pharmacology and Toxicology, College of Pharmacy, King Saud University, P.O. Box 2455, Riyadh 11451, Saudi Arabia; abdalshammari@ksu.edu.sa (A.A.); mesalharbi@ksu.edu.sa (M.A.); 2 Department of Pathology and Laboratory Medicine, Riyadh Security Forces Hospital, Ministry of Interior, Riyadh 11432, Saudi Arabia; amalghamdi@sfh.med.sa; 3 Ministry of Health, Riyadh 12613, Saudi Arabia; saif20_07@hotmail.com; 4 Department of Bioinformatics and Biotechnology, Government College University Faisalabad, Faisalabad 38000, Pakistan; usmancemb@gmail.com; 5 Department of Health and Biological Sciences, Abasyn University, Peshawar 25000, Pakistan; asadullahaup@gmail.com (A.U.); amjad.khan1@abasyn.edu.pk (A.K.); 6 Department of Oral Biology, College of Dentistry, University of Florida, Gainesville, FL 32611, USA; irfanmuhammad@ufl.edu

**Keywords:** antibiotic resistance, *Enterobacter xiangfangensis*, multi-epitope vaccine, molecular docking, molecular dynamics simulation

## Abstract

Antibiotic resistance is a global public health threat and is associated with high mortality due to antibiotics’ inability to treat bacterial infections. *Enterobacter xiangfangensis* is an emerging antibiotic-resistant bacterial pathogen from the Enterobacter genus and has the ability to acquire resistance to multiple antibiotic classes. Currently, there is no effective vaccine against Enterobacter species. In this study, a chimeric vaccine is designed comprising different epitopes screened from *E. xiangfangensis* proteomes using immunoinformatic and bioinformatic approaches. In the first phase, six fully sequenced proteomes were investigated by bacterial pan-genome analysis, which revealed that the pathogen consists of 21,996 core proteins, 3785 non-redundant proteins and 18,211 redundant proteins. The non-redundant proteins were considered for the vaccine target prioritization phase where different vaccine filters were applied. By doing so, two proteins; ferrichrome porin (FhuA) and peptidoglycan-associated lipoprotein (Pal) were shortlisted for epitope prediction. Based on properties of antigenicity, allergenicity, water solubility and DRB*0101 binding ability, three epitopes (GPAPTIAAKR, ATKTDTPIEK and RNNGTTAEI) were used in multi-epitope vaccine designing. The designed vaccine construct was analyzed in a docking study with immune cell receptors, which predicted the vaccine’s proper binding with said receptors. Molecular dynamics analysis revealed that the vaccine demonstrated stable binding dynamics, and binding free energy calculations further validated the docking results. In conclusion, these in silico results may help experimentalists in developing a vaccine against *E. xiangfangensis* in specific and Enterobacter in general.

## 1. Introduction

Antimicrobial resistance is a widespread public health problem; it affects the treatment of bacterial diseases, increases the hospital stay of patients and is linked to a higher rate of human mortality [1,2]. Antibiotic resistance is estimated to cause 33,000 deaths a year in the European countries alone [3]. Several bacterial species including *Escherichia coli*, *Salmonella typhi*, *Staphylococcus aureus*, *Clostridium burdile* and *Enterobacter* species have been seen to demonstrate resistance to several classes of antibiotics [2,4]. The misuse of antibiotics in our daily lives has applied selective pressure on bacterial cells and has led to the evolution of many drug-resistant strains of pathogenic bacteria, and now many antibiotics have lost their effectiveness [5,6]. Recommended measures such as the adaptation of antibiotic management programs and the improvement of diagnosis, follow-up and decision-making processes can make infectious diseases treatment process effective and reduce the dissemination of bacterial resistance. Increasing collaboration between stakeholders to develop new policies and investments to develop new antibacterial agents to combat bacterial pathogens is also promising [1,7]. New approved antibiotics and vaccines are expected to help in managing bacterial diseases and stop bacterial resistance to antibiotics [8,9].

*Enterobacter xiangfangensis* is a Gram-negative, motile, and 0.8–1 × 1–1.5 µm size bacteria [10,11]. *E. xiangfangensis* is involved in multiple hospital-acquired infections and shows high resistance to broad-spectrum antibiotics [12]. It is also reported that the bacteria has the ability to acquire carbapenemase genes from other bacterial species of the genus Enterobacter [11]. For *Enterobacter cloacae* complex (ECC), no appropriate vaccine is available, which makes the situation worse globally [13]. Vaccine use is an ideal approach for eradicating infectious diseases globally. Reverse vaccinology is a genomic-based technology to develop vaccines and has many advantages over traditional vaccine development such as the need for less cost and a small time period [14]. Genome-based reverse vaccinology has been utilized to develop a vaccine for *Neisseria meningitidis* serogroup B. Before that, there was no effective vaccine for *N. meningitidis* [15]. Once the said genome was sequenced, many unknown antigens were identified, which were then utilized to develop a vaccine. Thus, considering the good potential of reverse vaccinology in screening protective antigens from bacterial pathogens, herein, the technique is applied to *E. xiangfangensis* to support vaccine research against the targeted bacteria.

## 2. Research Methodology

**Figure 1:**
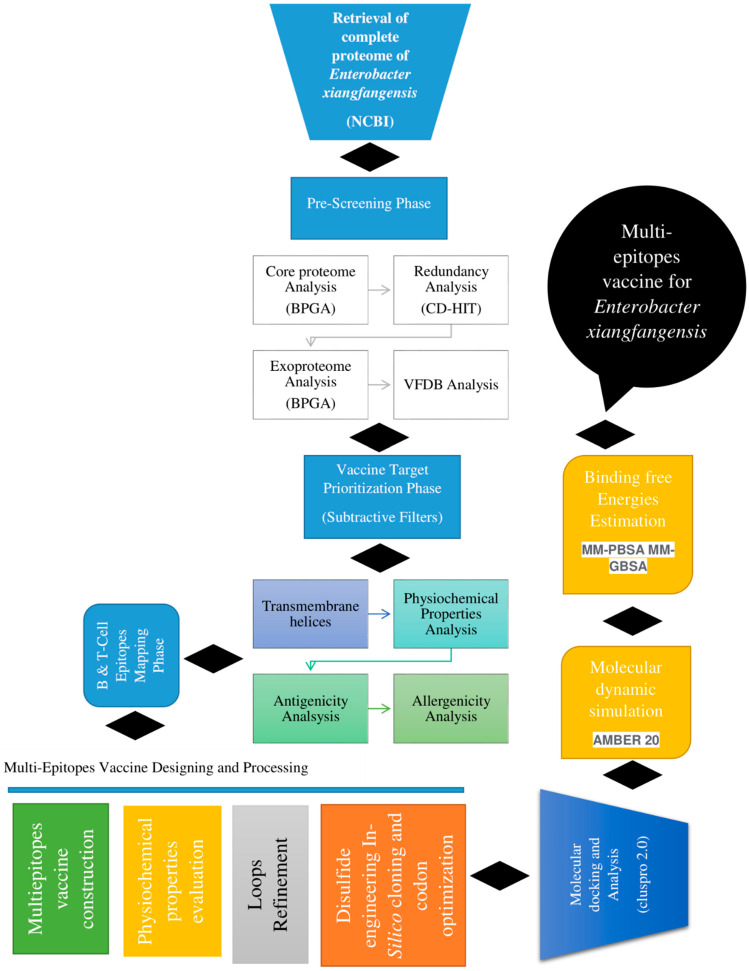
Schematic diagram of methods applied for designing a multi-epitope-based vaccine against *E. xiangfangensis*. The methods can be split into the retrieval of the complete proteome, prescreening phase, vaccine target prioritization phase, epitope prioritization and selection, multi-epitope vaccine designing and processing, molecular docking, molecular dynamics simulation and binding free energy calculations.

The overall flow diagram followed for the in silico design of a multi-epitope vaccine against *E. xiangfangensis* is presented in Figure 1.

### 2.1. Complete Proteome Retrieval and Bacterial Pan-Genome Analysis (BPGA)

The study began with the retrieval of the complete proteome of *E. xiangfangensis* from the National Center for Biotechnology Information (NCBI) database (https://www.ncbi.nlm.nih.gov/, accessed on 15 March 2022) followed by bacterial pan-genome analysis (BPGA). BPGA provides the core proteome of the pathogen, which is vital for broad-spectrum vaccine development [16]. In addition to core proteome sequences, pan-core and pan-phylogeny plots of the pathogen are also provided [16,17]. After the BPGA analysis, the core sequences were considered for further downward analysis.

### 2.2. Redundancy, Subcellular Localization and VFDB Analysis

Duplicated genes are paralogous genes and are mostly not required for vaccine development [18]. Hence, all of the redundant proteins were removed and non-redundant core sequences were extracted using the CD-HIT web server (http://weizhong-lab.ucsd.edu/cd-hit/, accessed on 15 March 2022) [19]. Surface-localized proteins are mostly exposed to the host immune system and are considered good vaccine targets [20]. To separate surface proteins, subcellular localization analysis was performed using the PSORTb tool (https://www.psort.org/psortb/, accessed on 15 March 2022) [21]. The cytoplasmic membrane and other proteins having multiple localization presences were discarded. Periplasmic membrane, outer membrane and extracellular membrane proteins were shortlisted for further studies. To check the virulency of surface-localized proteins, all the surface-localized proteins were subjected to virulence factor database (VFDB) [22] analysis available at http://www.mgc.ac.cn/VFs/, accessed on 15 March 2022. The selection criteria for virulence proteins were that the proteins must have >100 bit score and >30% sequence identities [13]. Virulent proteins stimulate strong immunological responses needed for a good vaccine [23].

### 2.3. Transmembrane Helices, Antigenicity, Allergenicity, Water Solubility and Physicochemical Property and Homology Analysis

Transmembrane helix analysis was performed using TMHMM-2.0 available at https://services.healthtech.dtu.dk/service.php?TMHMM-2.0, accessed on 16 March 2022, and proteins having more than 1 transmembrane helix were discarded [24,25]. Proteins with 0 or 1 transmembrane helix are easy to clone and express and thus were selected for further analysis [26]. Antigenicity analysis was performed using “VaxiJen 2.0” at http://www.ddg-pharmfac.net/vaxijen/VaxiJen/VaxiJen.html, accessed on 17 March 2022 [27]. A threshold of 0.6 was considered for the selection of vaccine proteins. Antigenic proteins stimulate strong immunological pathways and are regarded as good vaccine targets. Additionally, the allergenicity of the proteins was determined using the online Allertop 2.0 tool at https://www.ddg-pharmfac.net/AllerTOP/, accessed on 18 March 2022 [28]. The allergen sequences were removed, and the probable non-allergenic protein sequences were considered for water solubility and physicochemical property analysis. Water solubility was checked using the online web server of Innovagen at https://pepcalc.com/peptide-solubility-calculator.php, accessed on 19 March 2022. Physicochemical property analysis was performed using ProtParam Expassy at https://web.expasy.org/protparam/, accessed on 20 March 2022 [29]. Different types of physicochemical properties were assessed [30]. The proteins having stable physicochemical properties and good water solubility were next considered for homology analysis. The good vaccine candidates were further compared with human proteome (taxid: 9606) and human intestinal flora *Lactobacillus rhamnosus* (taxid: 47715), *L. johnsonii* (taxid: 33959) and *L. casei* (taxid: 1582) to discard host homologous and probiotic proteins to avoid autoimmune responses and accidental inhibition of probiotic bacteria, respectively [31]. This was achieved using the online BLASTp web server accessed at https://blast.ncbi.nlm.nih.gov/Blast.cgi, accessed on 22 March 2022 [32].

### 2.4. Epitope Prediction and Prioritization Phase

In the epitope selection and prioritization phase, linear B-cell epitopes were predicted using Bepipred Linear Epitope Prediction 2.0 on the IEDB web server at https://www.iedb.org/, accessed on 22 March 2022 [33,34]. The B-cell epitope prediction was revalidated by ABCpred (https://webs.iiitd.edu.in/raghava/abcpred/ABC_submission.html, accessed on 23 March 2022), which is an artificial neural network based B-cell prediction tool. T-cell epitopes were predicted using B-cell epitopes as input [35]. The T-cell epitopes were predicted both for MHC-I and MHC-II alleles using the full reference set of HLA alleles available at IEDB [20]. The T-cell epitope confirmation was performed using NetMHC-4.0 (https://services.healthtech.dtu.dk/service.php?NetMHC-4.0, accessed on 25 March 2022). Further, common epitopes were prioritized based on percentile score. A lower percentile score indicates a stronger binder. The antigenicity, allergenicity, water solubility and toxicity of good binders were evaluated using VaxiJen [20,27], Allertop 2.0 [28], Innovagen and Toxinpred (https://webs.iiitd.edu.in/raghava/toxinpred/algo.php, accessed on 28 March 2022), respectively. Additionally, the selected epitopes were also assessed for HLA-DRB1*0101 binding, and good HLA-DRB1*0101 binders were selected while the rest of the non-antigenic, probable allergic and poorly water-soluble epitopes were discarded [36].

### 2.5. Multi-Epitope-Based Vaccine Designing and Processing Phase

In multi-epitope designing and processing, a multi-epitope vaccine was constructed and then processed [37,38]. In the construction phase, the filtered epitopes were linked through “GPGPG” linkers and linked with “cholera toxin B-subunit adjuvant (CTBS)” via an EAAK linker [20,39,40]. The linkers used prevent epitopes from folding over one another and keep the epitopes separated so they can be easily presented to the host immune system [35]. After the construction of the vaccine construct, the physicochemical properties were analyzed for the designed vaccine construct using Protparam [41].

### 2.6. Structure Prediction and Loop Refinement

The 3D structure of the designed vaccine construct was modeled using the online Scratch Protein Predictor tool (http://scratch.proteomics.ics.uci.edu/, accessed on 1 April 2022) [42]. Ab initio modeling of the vaccine was performed due to the absence of a good 3D template. The loops of the designed vaccine and the vaccine structure were refined using GalaxyWEB services (https://galaxy.seoklab.org/, accessed on 2 April 2022) [43].

### 2.7. Disulfide Engineering and In Silico Codon Optimization Analysis

Disulfide engineering was performed to retain the stability of the designed vaccine construct using Designed 2.0 (http://cptweb.cpt.wayne.edu/DbD2/, accessed on 3 April 2022) [37]. The disulfide engineering was performed to prevent the degradation of the vaccine’s weak regions. In in silico codon optimization analysis, the designed vaccine construct was first converted to DNA sequences using the JCat tool (http://www.jcat.de/, accessed on 15 April 2022) [44], and then the DNA sequences were cloned in pET28a using the “SnapGene” tool (https://www.snapgene.com/, accessed on 15 April 2022).

### 2.8. Secondary Structure, Solubility, Z-Score and Population Coverage Analysis

The secondary structure of the designed vaccine was generated using the pdbsum generate tool (https://bio.tools/pdbsum_generate, accessed on 16 April 2022) [45]. The server also generates a Ramachandran plot. The solubility and Z-score of the designed vaccine were predicted using Protein Sol (https://protein-sol.manchester.ac.uk/, accessed on 16 April 2022) [46] and Prosa Web (https://prosa.services.came.sbg.ac.at/prosa.php, accessed on 16 April 2022) [47]. Moreover, population coverage analysis was performed in order to check world and country-wise coverage of the designed vaccine construct [48]. This was accomplished using the IEDB population coverage tool available at http://tools.iedb.org/population/, accessed on 16 April 2022. During the analysis, the final set of epitopes and their respective best binding alleles were used. The tool predicts the percentage of individuals who are likely to respond to the given set of epitopes with known HLA background. The calculation was performed by considering both class I and class II combined.

### 2.9. Molecular Docking

Molecular docking is an in silico approach where non-covalent interactions of the molecules such as proteins and ligands are predicted [49]. For docking analysis, first, different immune receptors such as MHC-I (pdb id: 1L1Y), MHC-II (pdb id: 1KG0) and TLR-4 (pdb id: 4G8A) were retrieved from Protein Data Bank, and the structures were prepared in UCSF Chimera 1.15 [50]. In the preparation phase, energy minimization was performed using the steepest descent and conjugate gradient algorithm for 750 steps. The Cluspro 2.0 online web server was utilized for docking purposes [51]. During docking, the chains of receptor molecules were specified for vaccine binding. Only a stable complex for each receptor was selected for visualization and dynamics studies. The selection of the top complex was performed based on the lowest binding energy in kcal/mol.

### 2.10. Molecular Dynamics Simulation

Molecular dynamics simulation is a computer-based approach that is mainly used to investigate the physical movement of docked complexes [52]. The ABMER 20 software package was used for molecular dynamics analysis [53]. The molecular dynamics simulation analysis was completed in three main steps: pre-processing, preparation and trajectory analysis. The preprocessing was performed using the Antechamber program [54]. The leap module of AMBER was used to record the topology of both receptors and vaccine molecules. The force field of FF14SB was employed for parameterization [55]. The energy optimization was performed using the steepest descent for 1000 steps and conjugate gradient for 1500 steps. The simulation time period was set at 200 ns. The temperature was maintained using Langevin dynamics. The CPPTRAJ module was considered for trajectory examination to check structure stability [56]. XMGRACE was used for creating different plots [57]. The intermolecular binding free energies were estimated using the MMGB-PBSA method by processing 1000 frames. MMGB-PBSA was run using the MMPBSA.py AMBER method [58,59].

### 2.11. Immune Simulation

To check the antibody and different immune responses of the host to the vaccine, an online C-ImmSim server was utilized [60]. The calculations were performed using the default parameters of the sever. The C-ImmSim server simulates three components of the human body, i.e., bone marrow, lymph node and thymus. The time step of injection was set to 1, while the number of adjuvant molecules added was 100. The number of antigens injected was 1000. The random seed value was 12,345, simulation volume was 10 and number of simulation steps was 100.

## 3. Results and Discussion

### 3.1. E. xiangfangensis Complete Proteome Retrieval and BPGA Analysis

**Figure 2:**
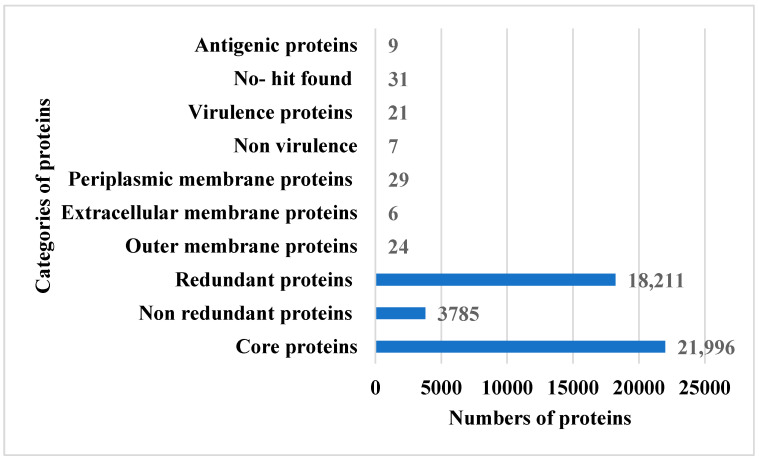
Different categories of proteins achieved in each step of proteome subtraction. It can be noticed that the size of the proteome is reduced by applying different filters, and only 9 appropriate antigenic proteins were prioritized as good subunit vaccine targets.

**Figure 3:**
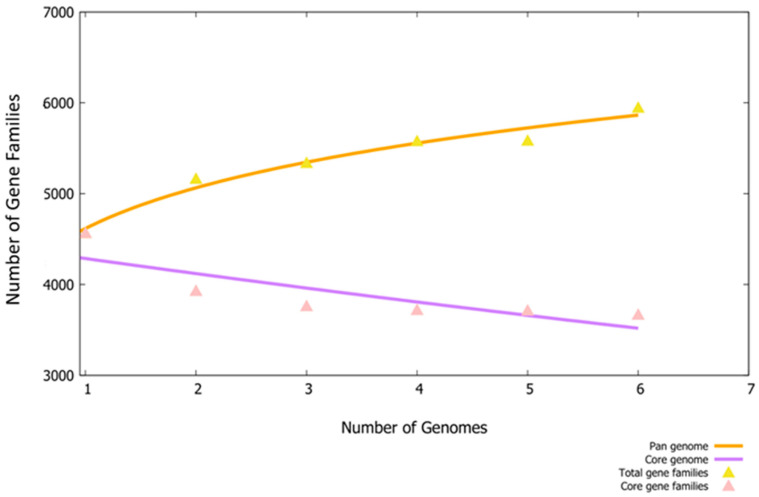
Pan-genome analysis of *E. xiangfangensis*. The plots demonstrate the number of gene families in each strain of the pathogen.

In this research study, total of six fully sequenced proteomes, namely (i) ASM80740v4, (ii) ASM81422v1, (iii) ASM396479v2, (iv) ASM399975v1, (v) ASM1493169v1 and (vi) ASM172978v1, were retrieved from the NCBI database. In the retrieval phase, several filters were applied (e.g., fully sequenced proteomes), humans were considered as hosts and incomplete proteomes were discarded. The BPGA pipeline was then utilized to extract core proteomes from pathogen complete proteomes. The BPGA results revealed that the pathogen consists of 21,996 core proteins, while the CD-HIT analysis revealed that the pathogen core proteins contain 3785 non-redundant proteins and 18,211 redundant proteins. The core proteins are regarded as good vaccine targets due to their major role in bacterial essential pathways and functionality [61]. The non-redundant proteins are duplicate copies of the pathogen genes and are considered bad vaccine candidates [62]. Subcellular localization analysis revealed that the 18,211 redundant proteins contain 24 outer membrane proteins, 6 extracellular membrane proteins and 29 periplasmic membrane proteins. The surface proteins are in direct contact with the host cells and have immune-dominant epitopes for activation of immune pathways; thus, they are good vaccine targets [63]. The VFDB analysis determined 7 as non-virulent while 21 were predicted as virulent and considered for further analysis. Virulent proteins activate strong infection and immunological pathways and therefore are attractive vaccine targets [23]. Antigenicity analysis predicted nine proteins as probable antigenic proteins. The number of proteins determined in each step of proteome subtraction is shown in Figure 2, while the size of the genome of each strain is shown in Figure 3.

**Figure 4:**
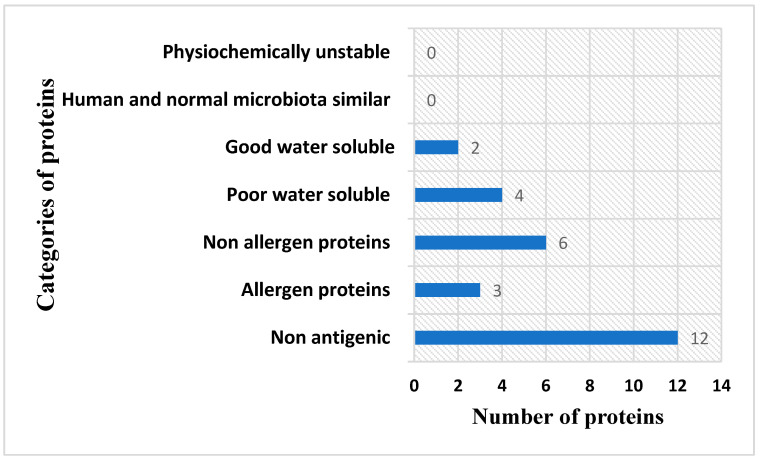
Vaccine target prioritization for epitope prediction. Different numbers of proteins filtered by different analyses.

Among the above-filtered sequences, no unstable proteins were predicted. Furthermore, no significant hits against human and probiotic bacteria were found, which ensures that autoimmune reactions will not be generated if the proteins are used in subunit vaccine designing [64]. Solubility analysis reported only two proteins as having good water solubility, and four were predicted as poorly water-soluble. The soluble proteins were further investigated for allergenicity, and six proteins were reported as non-allergens and three were predicted as allergens. The numbers of output proteins in all these analyses are presented in Figure 4.

### 3.2. Epitope Mapping Phase

In the epitope mapping phase, two proteins: ferrichrome porin (FhuA) and peptidoglycan-associated lipoprotein (Pal) were subjected to B-cell epitope prediction phase. From FhuA, eight B-cell epitopes were predicted, while from Pal, four epitopes were predicted, as tabulated in Table 1. The predicted B-cell epitopes were confirmed by ABCphred, which predicted most of Table 1 epitopes; however, some variation in the length and affinity was observed. These B-cell epitopes are vital in generating humoral immunity of the host and helping to create cellular immunity. Moreover, the predicted B-cell epitopes were considered for T-cell epitopes in the T-cell epitope prediction phase. Like B-cell epitopes, T-cell epitopes were validated by NetMHC-4.0. Both MHC-I and MHC-II epitopes were predicted, as mentioned in Table S1. The reference MHC alleles used are given in Tables S2 and S3. Only lower percentile score epitopes shared by both MHC classes were selected for further analysis. The predicted epitopes were further filtered in order to check antigenicity, allergenicity, DRB*0101 binding affinity, water solubility and toxicity. From the above screening, three epitopes, namely the GPAPTIAAKR, ATKTDTPIEK and RNNGTTAEI epitopes, were shortlisted and used in multi-epitope designing. These epitopes fulfill all good parameters for epitope-based vaccine design and are non-toxic.

### 3.3. Physicochemical Property Evaluation and Vaccine Structure Prediction 

**Figure 5:**
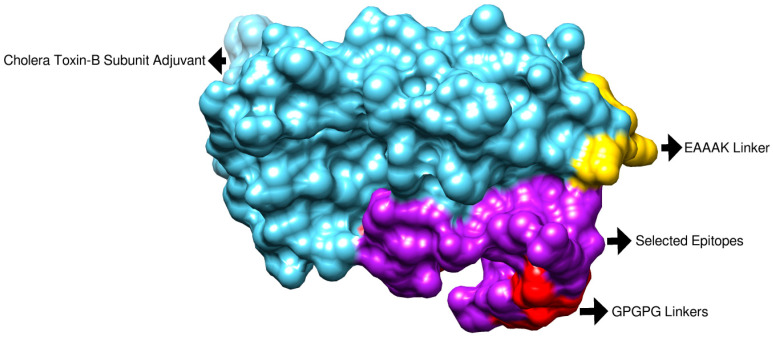
Three-dimensional (3D) structure of the designed vaccine construct. Each component of the vaccine is shown.

**Figure 6:**
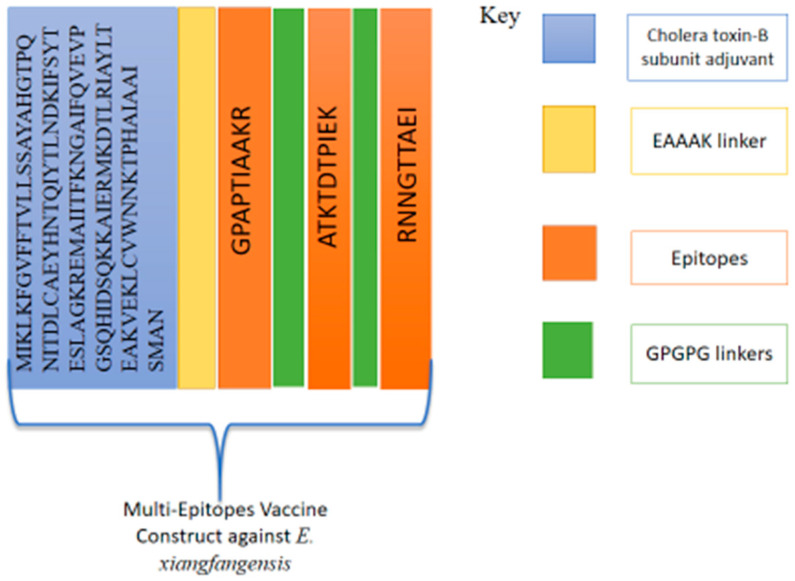
Schematic representation of multi-epitope vaccine construct for *E. xiangfangensis*.

Physicochemical property analysis predicted 168 amino acids for the vaccine. The vaccine’s molecular weight is 18.16 kDa, while the theoretical pI is 9.21. The estimated half-life is 30 h. The instability index (II) is computed to be 31.78; this classifies the protein as stable. The aliphatic index is 78.57 and the grand average of hydropathicity (GRAVY) is −0.277 for the final vaccine construct. The final vaccine construct also revealed an overall prediction for the protective antigen of 0.6534. The vaccine is a non-allergen, has good water solubility and has a good DRB*0101 binding score of an IC_50_ value (nM) less than 100 nM. The 3D structure was predicted using Scratch Protein Predictor, as shown in Figure 5. The vaccine is schematically presented in Figure 6. The multi-epitope vaccine is reported to show good immune responses compared to a single-epitope vaccine.

**Figure 7:**
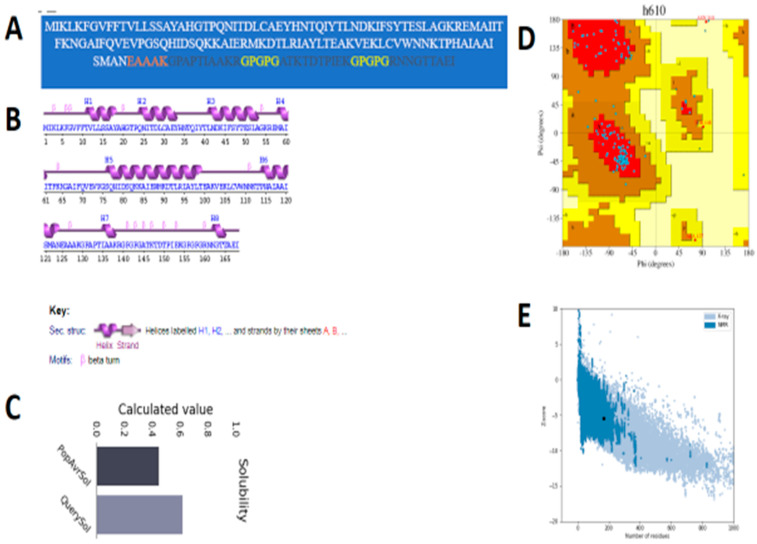
(**A**) Sequence of the vaccine construct. (**B**) Secondary structure. (**C**) Solubility prediction graph. (**D**) Ramachandran plot. (**E**) Z-score graph of the vaccine construct. The different color squares in the Ramachandran plot can be interpreted at the pdbsum generate website. The Z-score plot demonstrates the vaccine Z-score (represented by a black dot against blue and light blue shaded areas, which show the pdb structure of the same size).

The primary sequence of the vaccine is given in Figure 7A. Additionally, pdbsum was used to generate the predicted secondary structure and a Ramachandran plot for the vaccine. The secondary structure of the vaccine showed 78 (46.4%) alpha helices (Figure 7B). There were 130 (90.9%) most favored region residues, 10 (7.0%) additional allowed region residues, 2 (1.4%) generously allowed region residues, 1 (0.7%) disallowed region residue and 143 (100.0%) non-glycine and non-proline residues. The vaccine is a molecule with good water solubility (predicted scaled solubility: 0.617) (Figure 7C). Moreover, there were 2 end-residues (excluding Gly and Pro), 13 glycine residues and 110 proline residues, and the total number of residues was 168, as shown in Figure 7D. The Z-score of the vaccine is −5.47, as shown in Figure 7E.

### 3.4. Loop Refinement, Disulfide Engineering, In Silico Codon Optimization and Population Coverage Analysis

**Figure 8:**
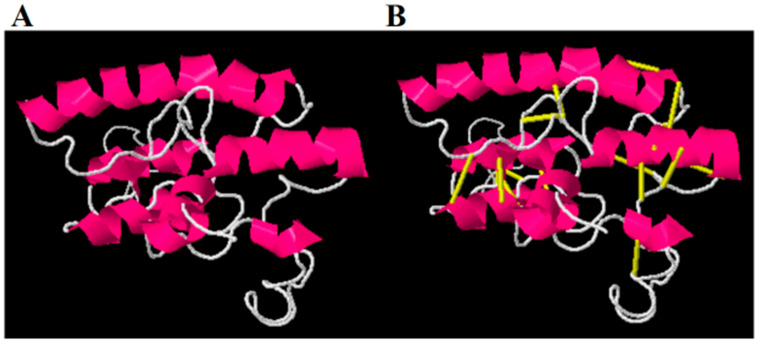
Wild structure of the vaccine (**A**) and mutated structure of the vaccine (**B**). The yellow sticks represent replaced amino acids.

**Figure 9:**
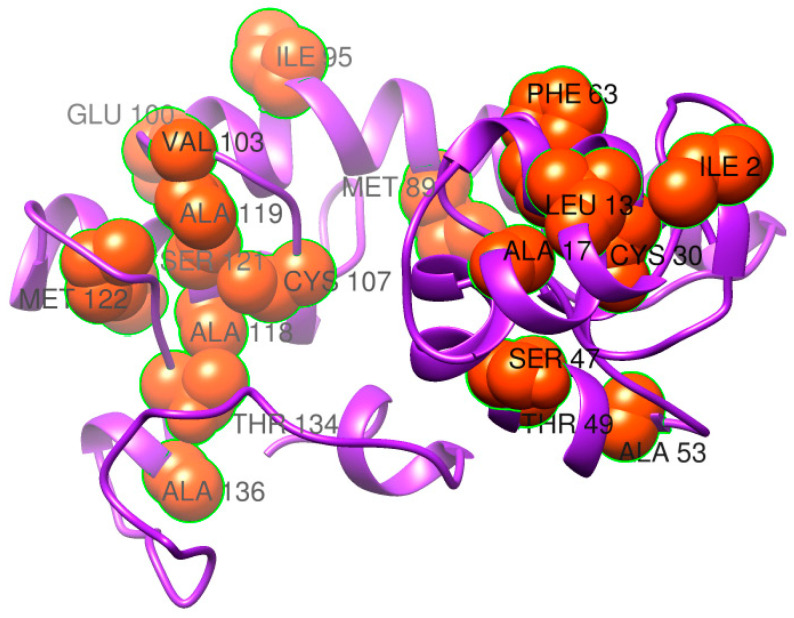
The 3D structure of the mutant vaccine with amino acids having high unstable energy in kcal/mol selected for disulfide engineering.

To retain the rigidity of the vaccine and remove structure errors from the vaccine structure, refinement was performed, and the results are mentioned in Table 2. The model 1 vaccine has better structural properties compared to the rest of the structures. Disulfide engineering was performed and replaced all the amino acid residues that are sensitive to enzymatic degradation [65]. The targeted residues were replaced by cysteine amino acid as represented by yellow sticks in the mutated structure in Figure 8. Additionally, the replaced amino acids are also represented by spheres in the 3D structure shown in Figure 9. The pairs of amino acid residues and their chi3 energy value obtained during disulfide engineering are tabulated in Table S4.

**Figure 10:**
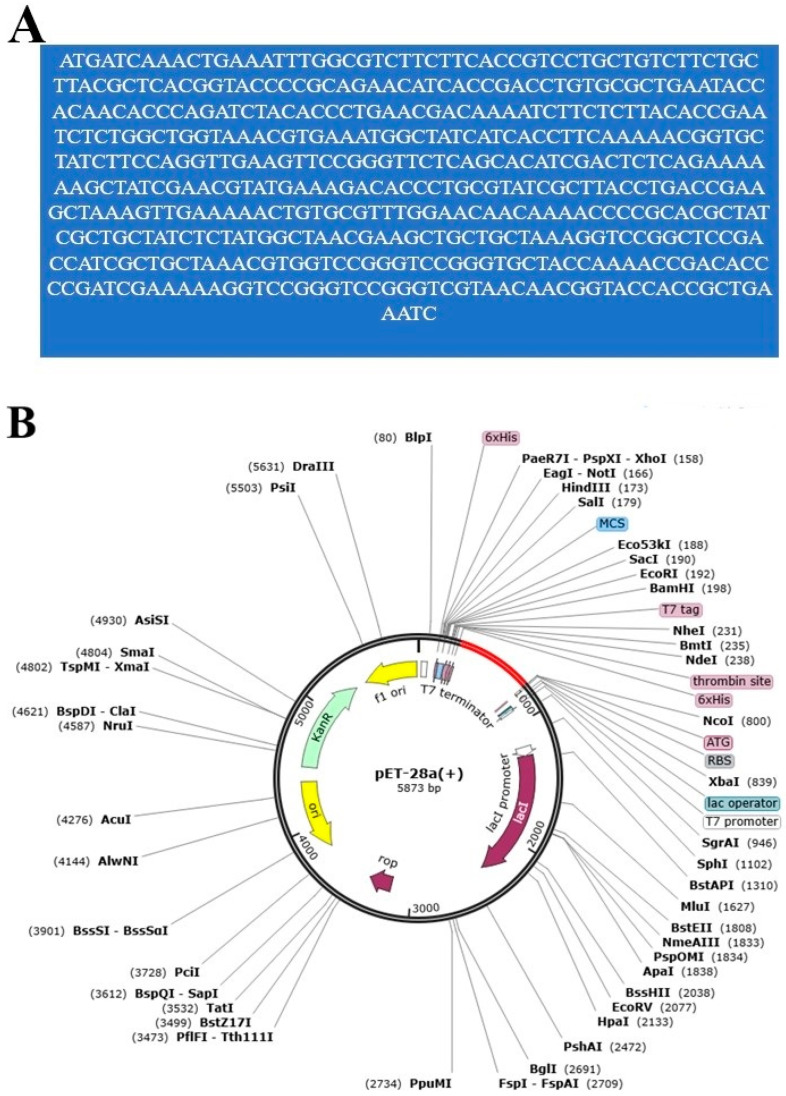
Codon optimization and cloning analysis of vaccine. (**A**) DNA sequence of the vaccine (**B**) In silico cloned pET28a (+) vector.

In in silico codon optimization, the vaccine was converted to the DNA sequence “ATGATCAAACTGAAATTTGGCGTCTTCTTCACCGTCCTGCTGTCTTCTGCTTACGCTCACGGTACCCCGCAGAACATCACCGACCTGTGCGCTGAATACCACAACACCCAGATCTACACCCTGAACGACAAAATCTTCTCTTACACCGAATCTCTGGCTGGTAAACGTGAAATGGCTATCATCACCTTCAAAAACGGTGCTATCTTCCAGGTTGAAGTTCCGGGTTCTCAGCACATCGACTCTCAGAAAAAAGCTATCGAACGTATGAAAGACACCCTGCGTATCGCTTACCTGACCGAAGCTAAAGTTGAAAAACTGTGCGTTTGGAACAACAAAACCCCGCACGCTATCGCTGCTATCTCTATGGCTAACGAAGCTGCTGCTAAAGGTCCGGCTCCGACCATCGCTGCTAAACGTGGTCCGGGTCCGGGTGCTACCAAAACCGACACCCCGATCGAAAAAGGTCCGGGTCCGGGTCGTAACAACGGTACCACCGCTGAAATC” and then inserted into the pET28a (+) vector as shown by red color after 6x histidine. The primary nucleotide sequence of vaccine is provided in Figure 10A, while the inserted DNA sequence in the vector is given in Figure 10B. The CAI value of the vaccine is 0.95, and the GC score has a value of 50.79. These values specify high expression of the vaccine if cloned in the same vector and expressed in the *Escherichia coli* K12 strain [66].

**Figure 11:**
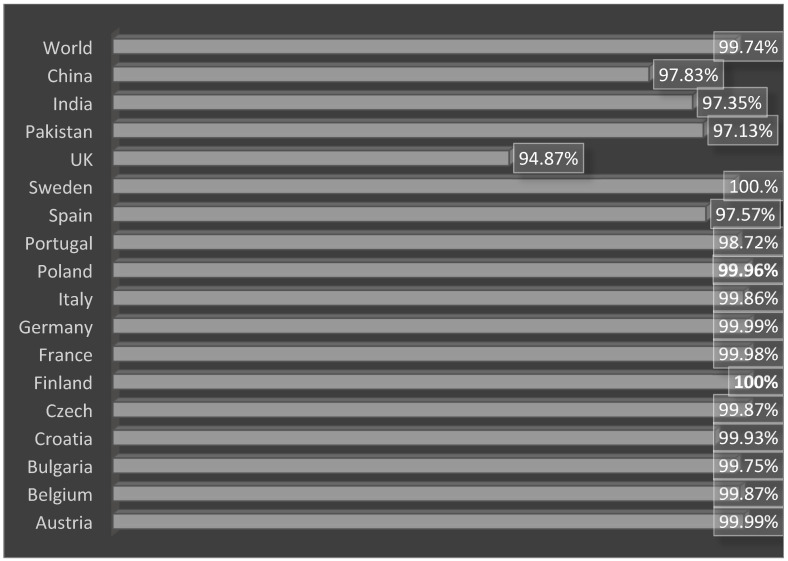
Population coverage percentages of the designed vaccine across different countries.

In population coverage analysis, the vaccine construct was checked for world and country-wise coverage. According to IEDB population coverage, the designed vaccine construct molecule was able to cover a worldwide population of 99.74%, while the highest countrywide populations are in China (97.83%), India (97.35%) and Pakistan (97.13%). The vaccine construct has the ability to provide immunity against the pathogen in 100% of the population of Sweden, as shown in Figure 11. For more data, please refer to Table S5.

### 3.5. Molecular Docking Analysis

**Figure 12:**
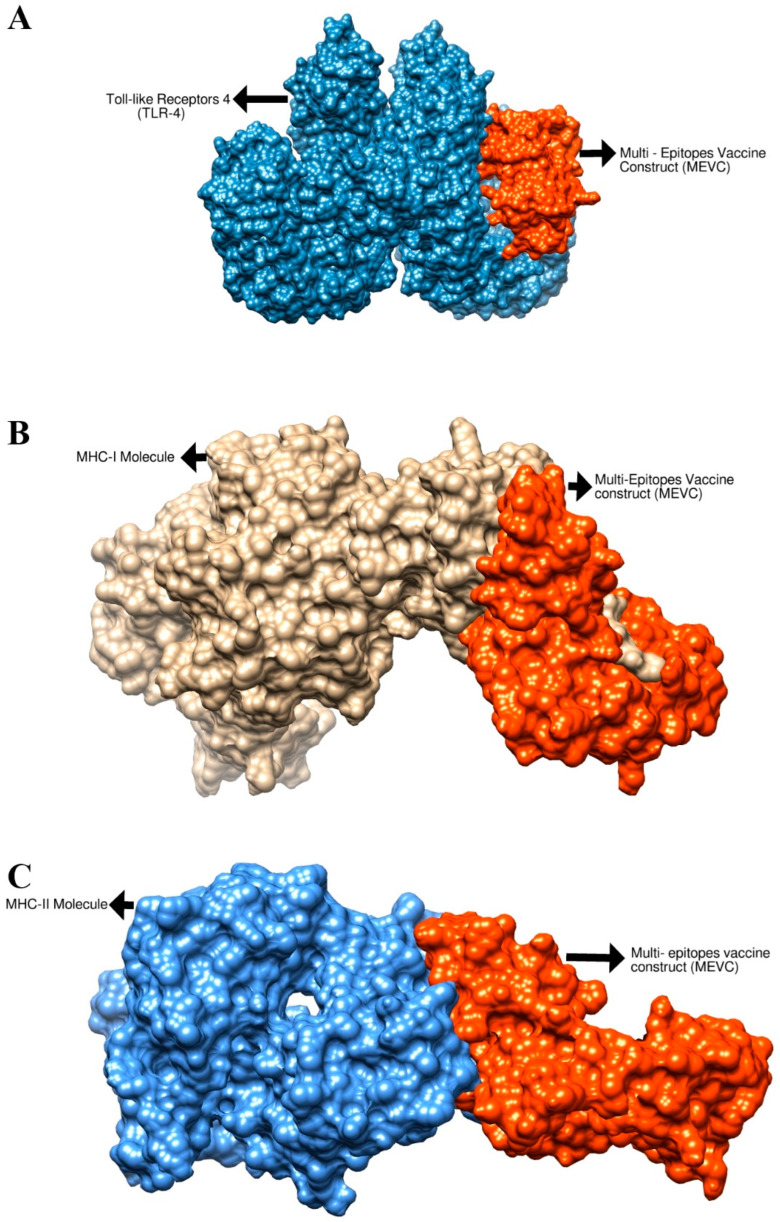
Structure of the vaccine docked to the TLR-4 molecule (**A**), MHC-I molecule (**B**) and MHC-II molecule (**C**). The receptors are represented by colored surfaces while the vaccine is represented by an orange surface.

Molecular docking analysis was performed to check the binding affinity of the vaccine construct to immune cell receptors MHC-I, MHC-II and TLR-4. For docking purposes, Cluspro2.0 was utilized [67]; in each case, the top 10 docking solutions were generated. The results were interpreted through binding energy; the solution which has the lowest binding energy value demonstrates stronger intermolecular binding affinity. The docked complexes which have the lowest binding energy were selected for interaction visualization analysis. All top 10 generated docked solutions for each receptor and their binding energy are tabulated in Tables S6–S8. The best docked solution in each case was considered for molecular dynamics analysis. The intermolecularly docked complexes are presented in 3D structure in Figure 12. The binding energy value of the vaccine with MHC-, MHC-II and TLR-4 is −733.6 kcal/mol, −696.0 kcal/mol and −691.7 kcal/mol, respectively. In these stable complexes, it was observed that the vaccine binding mode with the immune receptor is deep and interactions are dominated by close-distance van der Waals forces and hydrogen bonds. Moreover, it was noticed that the epitopes are exposed, which ensures that they can be easily recognized and processed by immune cells. The interaction residues between the vaccine and immune receptors were examined within 5 Å, which revealed a rich interaction profile including both hydrophilic and hydrophobic contacts as given in Table 3.

### 3.6. Molecular Dynamics Simulation (MDS) and Binding Free Energy Calculation

**Figure 13:**
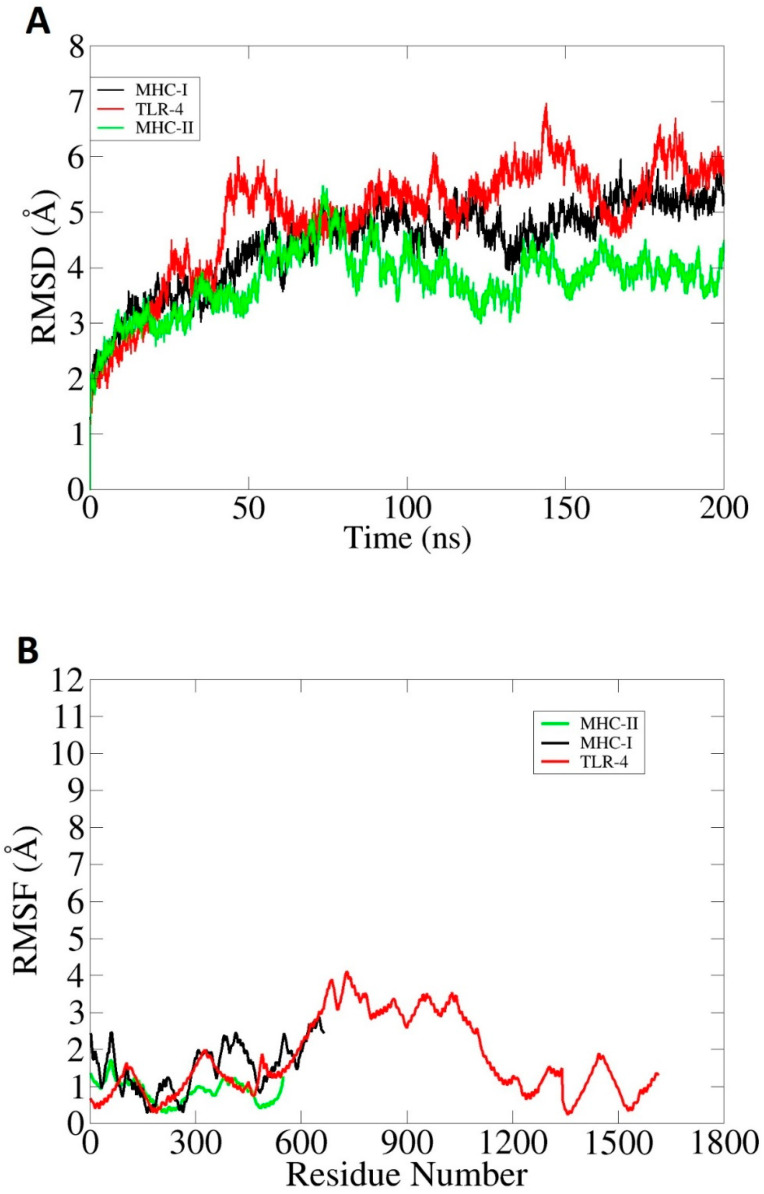
Graphical representation of simulation trajectories: (**A**) RMSD; (**B**) RMSF. These analyses were performed based on carbon alpha atoms.

MDS analysis is an in silico approach for evaluating the dynamic behavior of docked molecules. The simulation analysis consists of (i) root mean square deviation (RMSD) [68] and (ii) root mean square fluctuation (RMSF) [69]. The RMSD allows the superimposition of all simulation snapshots based on carbon alpha atoms. The deviation was measured in the term of angstroms and schemed as shown in Figure 13A. The complexes were found to have good stability, and the RMSD was found to be within 7 Å. The vaccine–MHC-II complex was highly stable with very minor fluctuations. The RMSD of the vaccine–MHC-I was seen to exhibit an increasing trend, but in the end, the graph became stable and no drastic changes were observed further. Throughout the length of simulation time, minor structure variations can be noticed, which is understandable considering the large interacting surface area of the molecules and the high percentage of loops in the receptors. However, it was noticed the intermolecular interactions are strong enough to keep the binding mode stable. Next, the docked complexes were analyzed on the residue level of fluctuations via the RMSF. In RMSF analysis, very low-level fluctuations were observed throughout the simulation time for each docked complex. Little fluctuations might be due to vaccine adjustment at the docked site. However, these fluctuations did not affect the overall stability and binding mode of the vaccine to receptors as shown in Figure 13B. The results of the simulation were further validated through binding free energy calculations. This was achieved through MM-PBSA and MM-GBSA analysis. The MM-GBSA findings revealed the net delta energies of −225.97 kcal/mol for the vaccine–TLR-4 complex, −181.99 kcal/mol for the vaccine–MHC-I complex and 177.52 kcal/mol for the vaccine–MHC-II complex. Moreover, in MM-PBSA analysis, net energies of −231.98 kcal/mol for vaccine–TLR-4 complex, −189.9 kcal/mol for vaccine–MHC-I complex and −177.43 kcal/mol for vaccine–MHC-II complex were estimated, as mentioned in Table 4. Generally, the free binding energy estimations reported the good overall stability of the systems.

### 3.7. Immune Simulation for Model Vaccine

**Figure 14:**
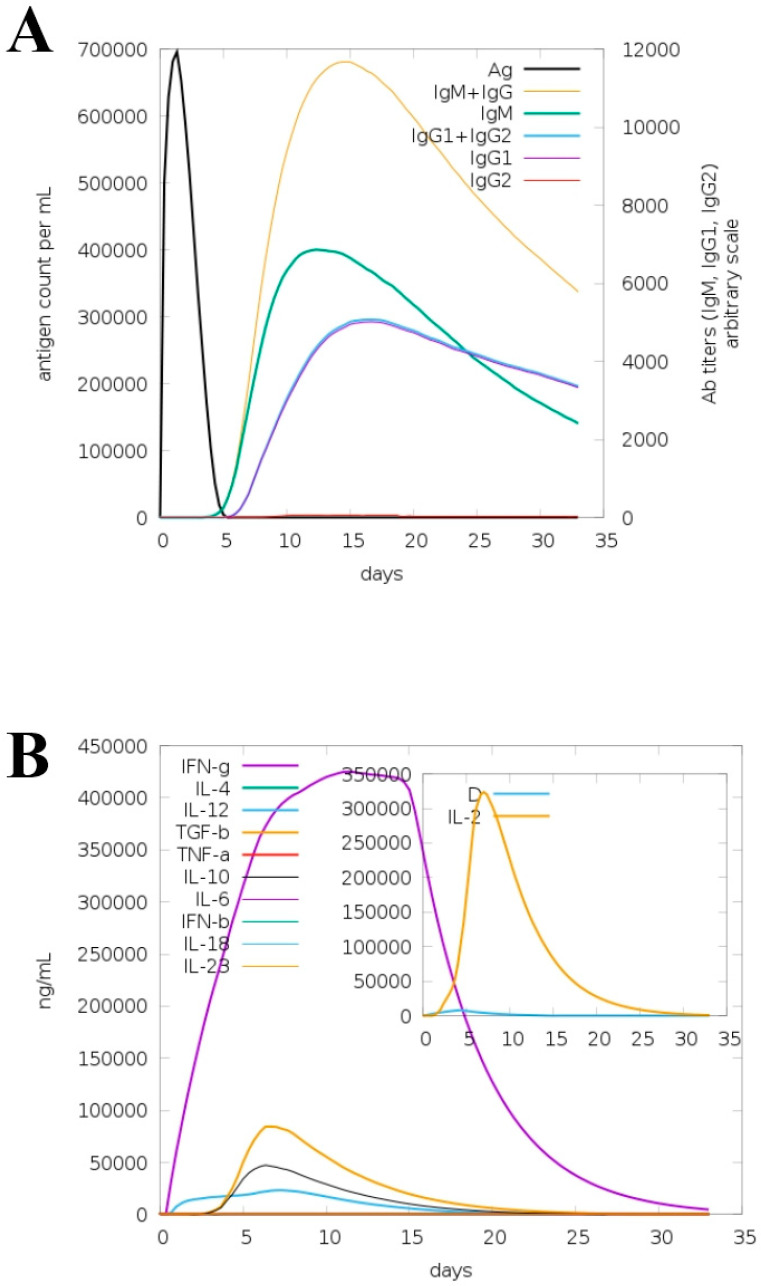
Host immune responses to the designed vaccine construct. Immunoglobulin responses to the vaccine (**A**); interferon and cytokine responses to the vaccine (**B**).

The “C-IMMSIM” server was utilized to check the immunogenic profile of the model vaccine for a period of about 35 days. The server predicted primary as well as secondary immune responses to the vaccine in the form of different types of antibodies. Additionally, the combination of IgM and IgG was also seen in the higher titers, followed by “IgG1 + IgG2” and IgM, as shown in Figure 14A. Different types of cytokines and interleukins were also observed in response to the vaccine, as shown in Figure 14B. Among them, IFN-g, TGF-a, IL-6 and IL-4 are the most promising responses against the vaccine. The elevated antibody rate and different types of cytokines demonstrated that the vaccine molecules can properly induce host immune responses. It further implies that both humoral immunity and cell-mediated immunity are key in the clearance of the antigen.

## 4. Conclusions

Currently, no FDA-approved vaccines are available against *E. xiangfangensis* and other members of the genus Enterobacter, though several are under clinical investigation. In the present study, a chimeric multi-epitope vaccine construct was designed to tackle *E. xiangfangensis* infections by considering all sequenced strains of the said pathogen. Two proteins, FhuA and Pal, were shortlisted as good vaccine candidates and harbored both B- and T-cells epitopes. The predicted epitopes were checked for antigenicity, allergenicity, water solubility and toxicity, and only three epitopes were predicted as probable antigen, non-allergen, non-toxic, DRB*0101 binders with good water solubility. The designed vaccine construct was subjected to molecular docking study with immune cell receptors, which predicted that the designed vaccine has the ability to interact with said immune cell receptors and can evoke both humoral and cellular immunity as demonstrated by C-immune simulation. Additionally, the molecular dynamics simulation analysis revealed that the intermolecular interactions between vaccine construct and immune cell receptors are quite stable. Regardless of the promising results, experimental validations are still needed to determine the real immune protection ability of the vaccine. 

## Figures and Tables

**Table 1:** Predicted B-cell epitopes from shortlisted vaccine targets.

Vaccine Candidate Protein	B-Cell Peptide	Antigenicity Score
core/255/1/Org1_Gene3420 (ferrichrome porin (FhuA))	AAETPKKEETITVTAAPAAQESAWGPAPTIAAKRTATATKTDTPIEKTPQSISVVTREEMDMKQPGT	0.78
	PTTEPLKEIQFKMGTDNLWQTGFD	0.53
	LPREGTVVPYYDANGKAHKLPTDFNEGDEDNKISRR	0.98
	NDTFTVRQNLRYTK	0.45
	TSAFNRNNGTTAEINDQAF	0.62
	FEPLSGTTQGGKPFD	0.42
	TADPANPTSGFSVQG	0.52
	NTVTYYSSASPKAYESFNV	0.85
core/4064/1/Org1_Gene715 (peptidoglycan-associated lipoprotein (Pal))	SNKNASNDQSGEGMMGAGTGMDANGNGNMSSEEQARLQMQQLQQNNIVYFDLDKYDIRS	0.42
	DERGTPEYNISL	0.40
	SYGKEKPAVLGHDEAAYSKN	0.63
	SNKNASNDQSGEGMMGAGTGMDANGNGNMSSEEQARLQMQQLQQNNIVYFDLDKYDIRS	0.71

**Table 2:** Structural features of top 10 models generated after refining multi-epitope vaccine structure.

Model	RMSD	MolProbity	Clash Score	Poor Rotamers	Rama Favored	GALAXY Energy
Initial	0.000	4.112	237.6	7.9	88.0	32,580.03
Model 1	1.055	1.757	5.5	0.0	92.8	−2764.21
Model 2	0.945	1.785	6.9	0.0	94.0	−2752.33
Model 3	1.115	1.884	9.0	0.0	94.0	−2750.91
Model 4	2.568	1.683	4.5	0.7	92.8	−2737.98
Model 5	1.068	1.857	7.3	0.0	92.8	−2734.97
Model 6	2.477	1.709	5.2	0.7	93.4	−2729.57
Model 7	1.017	1.779	5.2	0.0	91.6	−2718.37
Model 8	2.229	1.679	4.2	0.0	92.2	−2718.16
Model 9	0.933	1.870	8.7	0.7	94.0	−2717.94
Model 10	2.437	1.683	4.5	0.7	92.8	−2716.26

**Table 3:** Vaccine–immune receptor interacting residues within 5 Å.

Complex	Interacting Residues
Vaccine–MHC-I Complex	Asp66, Ala173, Asn312, Ala74, Phe40, Ala68, Val119, Gly121, Thr80, Glu35, Gln34, Arg4, Asp17, Val44, Asn60, Arg105, Val44, Asn103, Pro187, Arg72, Glu17, Val42, Ile31, Phe122
Vaccine–MHC-II Complex	Asn42, Lys41, Gly43, Glu44, Arg45, Lys94, Ala14, Arg97, Glu16, Asp98, Arg273, Tyr257, Leu272, Thr258, Arg219, Asp223, Gln222, Pro232, Phe208, Gln224, Lys19, Thr240, Ser61, Gly239, Val231 Arg202, His192
Vaccine–TLR-4 Complex	Asn526, Leu25, Asp502, Lys477, Glu272, Tyr451, Phe573, Val605, Ser582, Ile450, Gln578, Val548, Ser552, Leu548

**Table 4:** Estimation of binding free energies in kcal/mol by MM-GBSA and MM-PBSA methods.

Energy Parameter	TLR-4–Vaccine Complex	MHC-I–Vaccine Complex	MHC-II–Vaccine Complex
MM-GBSA			
VDWAALS	−190.74	−180.60	−168.55
EEL	−90.23	−54.87	−60.97
Delta G gas	−280.97	−235.47	−229.52
Delta G solv	55.00	53.48	52.00
Delta Total	−225.97	−181.99	177.52
MM-PBSA			
VDWAALS	−190.74	−180.60	−168.55
EEL	−90.23	−54.87	−60.97
Delta G gas	−280.97	−235.47	−229.52
Delta G solv	48.99	45.57	52.09
Delta Total	−231.98	−189.9	−177.43

## Data Availability

The data presented in this study are available within the article.

## Supplementary Materials

The following supporting information can be downloaded at: https://www.mdpi.com/article/10.3390/ijerph19137723/s1, Table S1: T-cells epitopes for the B-cell epitopes; Table S2: Allele for major histocompatibility complex-I; Table S3: Allele for Major histocompatibility complex-II; Table S4: Pairs of amino acid residues opted for disulfide engineering with Chi3 energy in kal/mol and sum B-factors; Table S5: Population coverage analysis of vaccine epitopes; Table S6: Docking score of vaccine with MHC-I; Table S7: Docking score of vaccine with MHC-II; Table S8: Docking score of vaccine with TLR-4.
