# Supplementary material for: Computer-Aided Multi-Epitope Vaccine Design against Enterobacter xiangfangensis
Source: Int J Environ Res Public Health. 2022 Jun 23;19(13):7723. doi: 10.3390/ijerph19137723 (PMC9265868; doi:10.3390/ijerph19137723)
Supplement: Supplementary file 1 [file ijerph-19-07723-s001.zip › ijerph-1705094-supplementary.pdf]

# Supplementary Files

## Computer Aided Multi-Epitopes Vaccine Design against *Enterobacter Xiangfangensis*

**Abdulrahman Alshammari[1], Metab Alharbi[1], Abdullah Alghamdi[2], Saif Ali Alharbi[3], Usman Ali Ashfaq[4], Muhammad Tahir ul Qamar[4,*], Asad Ullah[5], Muhammad Irfan[6], Amjad khan[5], Sajjad Ahmad[5,*]**

[1]Department of Pharmacology and Toxicology, College of Pharmacy, King Saud University, Post Box 2455, Riyadh, 11451, Saudi Arabia; Abdalshammari@ksu.edu.sa (A.A.); mesalharbi@ksu.edu.sa (M.A.)
[2]Department of Pathology and Laboratory Medicine, Riyadh Security Forces Hospital, Ministry of Interior, kingdom of Saudi Arabia; malghamdi@sfh.med.sa
[3]Ministry of Health, Kingdom of Saudi Arabia, Riyadh, Saudi Arabia; saif20_07@hotmail.com
[4]Department of Bioinformatics and Biotechnology, Government College University Faisalabad, Faisalabad 38000, Pakistan; usmancemb@gmail.com (U.A.A.); tahirulqamar@gcuf.edu.pk (M.T.Q.)
[5]Department of Health and Biological Sciences, Abasyn University, Peshawar 25000, Pakistan;
asadullahaup@gmail.com (A.U.); sajjad.ahmad@abasyn.edu.pk (S.A.); Amjad.khan1@abasyn.edu.pk (A.K.)
[6]Department of Oral Biology, College of Dentistry, University of Florida, Gainesville, FL 32611, USA; irfanmuhammad@ufl.edu
* Correspondence: (M.T.Q.); sajjad.ahmad@abasyn.edu.pk (S.A.)

**Table S1.** T-cells epitopes for the B-cell epitopes.

<table>
<tr><th>MHC-I</th><th>Percentile score</th><th>MHC-II</th><th>Percentile score</th></tr>
<tr><td>ETITVTAAPA</td><td>0.15</td><td rowspan="2">EETITVTAAPAAQES</td><td rowspan="2">0.36</td></tr>
<tr><td>TAAPAAQES</td><td>3.4</td></tr>
<tr><td>GPAPTIAAKR</td><td>0.03</td><td>AWGPAPTIAAKRTATA</td><td>4.1</td></tr>
<tr><td>ATKTDTPIEK</td><td>0.02</td><td>TATKTDTPIEKTPQ</td><td>0.39</td></tr>
<tr><td>VTREEMDMK</td><td>0.46</td><td rowspan="2">QSISVVTREEMDMKQPGT</td><td rowspan="2">8.01</td></tr>
<tr><td>QSISVVTRE</td><td>4.3</td></tr>
<tr><td>TEPLKEIQF</td><td>0.31</td><td>TTEPLKEIQFKMGT</td><td>9.2</td></tr>
<tr><td>QFKMGTDNLW</td><td>1.2</td><td>QFKMGTDNLWQTGFD</td><td>20</td></tr>
<tr><td>VVPYYDANGK</td><td>0.89</td><td>EGTVVPYYDANGKAH</td><td>9.9</td></tr>
<tr><td>EGDEDNKISR</td><td>2.5</td><td>TDFNEGDEDNKISRR</td><td>36</td></tr>
<tr><td>VVPYYDANGK</td><td>0.89</td><td>GTVVPYYDANGK</td><td>6.2</td></tr>
<tr><td>EGDEDNKISR</td><td>2</td><td>FNEGDEDNKISR</td><td>20</td></tr>
<tr><td>RNNGTTAEI</td><td>2.9</td><td>SAFNRNNGTTAEI</td><td>0.94</td></tr>
<tr><td>TTAEINDQA</td><td>0.06</td><td>NNGTTAEINDQAF</td><td>9.1</td></tr>
<tr><td>RNNGTTAEI</td><td>2.9</td><td>AFNRNNGTTAEI</td><td>1.1</td></tr>
<tr><td>RNNGTTAEI</td><td>2.9</td><td>NGTTAEINDQAF</td><td>7.5</td></tr>
<tr><td>ADPANPTSGF</td><td>13</td><td>ADPANPTSGFSV</td><td>22</td></tr>
<tr><td>ADPANPTSGF</td><td>0.13</td><td>VTYYSSASPKA</td><td>0.29</td></tr>
<tr><td>ASPKAYESF</td><td>0.28</td><td>ASPKAYESFNV</td><td>11</td></tr>
<tr><td>QARLQMQQL</td><td>0.12</td><td rowspan="2">EQARLQMQQLQQNNI</td><td rowspan="2">0.06</td></tr>
<tr><td>MQQLQQNNI</td><td>2.3</td></tr>
<tr><td>IVYFDLDKY</td><td>0.15</td><td rowspan="2">NNIVYFDLDKYDIRS</td><td rowspan="2">0.71</td></tr>
<tr><td>DLDKYDIRS</td><td>6.5</td></tr>
<tr><td>GMDANGNGNM</td><td>6.7</td><td>GTGMDANGNGNMSSE</td><td>28</td></tr>
<tr><td>ASNDQSGEGM</td><td>1.5</td><td>ASNDQSGEGMMGAGT</td><td>14</td></tr>
<tr><td>GTPEYNISL</td><td>0.51</td><td>ERGTPEYNISL</td><td>50</td></tr>
<tr><td>AVLGHDEAAY</td><td>0.28</td><td>KPAVLGHDEAAYSKN</td><td>9.1</td></tr>
<tr><td>YGKEKPAVL</td><td>0.01</td><td>SYGKEKPAVLGHDEA</td><td>34</td></tr>
</table>

**Table S2.** Allele for major histocompatibility complex- I.

| **Allele** | **Length** |
|---|---|
| HLA-A*01:01 | 9 |
| HLA-A*01:01 | 10 |
| HLA-A*02:01 | 9 |
| HLA-A*02:01 | 10 |
| HLA-A*02:03 | 9 |
| HLA-A*02:03 | 10 |
| HLA-A*02:06 | 9 |
| HLA-A*02:06 | 10 |
| HLA-A*03:01 | 9 |
| HLA-A*03:01 | 10 |
| HLA-A*11:01 | 9 |
| HLA-A*11:01 | 10 |
| HLA-A*23:01 | 9 |
| HLA-A*23:01 | 10 |
| HLA-A*24:02 | 9 |
| HLA-A*24:02 | 10 |
| HLA-A*26:01 | 9 |
| HLA-A*26:01 | 10 |
| HLA-A*30:01 | 9 |
| HLA-A*30:01 | 10 |
| HLA-A*30:02 | 9 |
| HLA-A*30:02 | 10 |
| HLA-A*31:01 | 9 |
| HLA-A*31:01 | 10 |
| HLA-A*32:01 | 9 |
| HLA-A*32:01 | 10 |
| HLA-A*33:01 | 9 |

| | |
|---|---|
| HLA-A*33:01 | 10 |
| HLA-A*68:01 | 9 |
| HLA-A*68:01 | 10 |
| HLA-A*68:02 | 9 |
| HLA-A*68:02 | 10 |
| HLA-B*07:02 | 9 |
| HLA-B*07:02 | 10 |
| HLA-B*08:01 | 9 |
| HLA-B*08:01 | 10 |
| HLA-B*15:01 | 9 |
| HLA-B*15:01 | 10 |
| HLA-B*35:01 | 9 |
| HLA-B*35:01 | 10 |
| HLA-B*40:01 | 9 |
| HLA-B*40:01 | 10 |
| HLA-B*44:02 | 9 |
| HLA-B*44:02 | 10 |
| HLA-B*44:03 | 9 |
| HLA-B*44:03 | 10 |
| HLA-B*51:01 | 9 |
| HLA-B*51:01 | 10 |
| HLA-B*53:01 | 9 |
| HLA-B*53:01 | 10 |
| HLA-B*57:01 | 9 |
| HLA-B*57:01 | 10 |
| HLA-B*58:01 | 9 |
| HLA-B*58:01 | 10 |

**Table S3.** Allele for Major histocompatibility complex- II**.**

HLA-DRB1*01:01

HLA-DRB1*03:01

HLA-DRB1*04:01

HLA-DRB1*04:05

HLA-DRB1*07:01

HLA-DRB1*08:02

HLA-DRB1*09:01

HLA-DRB1*11:01

HLA-DRB1*12:01

HLA-DRB1*13:02

HLA-DRB1*15:01

HLA-DRB3*01:01

HLA-DRB3*02:02

HLA-DRB4*01:01

HLA-DRB5*01:01

HLA-DQA1*05:01/DQB1*02:01

HLA-DQA1*05:01/DQB1*03:01

HLA-DQA1*03:01/DQB1*03:02

HLA-DQA1*04:01/DQB1*04:02

HLA-DQA1*01:01/DQB1*05:01

HLA-DQA1*01:02/DQB1*06:02

HLA-DPA1*02:01/DPB1*01:01

HLA-DPA1*01:03/DPB1*02:01

HLA-DPA1*01:03/DPB1*04:01

HLA-DPA1*03:01/DPB1*04:02

HLA-DPA1*02:01/DPB1*05:01

HLA-DPA1*02:01/DPB1*14:01

**Table S4.** Pairs of amino acid residues opted for disulfide engineering with Chi3 energy in kal/mol and sum B-factors.

| Amino acid pair | Chi3 | Energy | Sum B-Factors |
|---|---|---|---|
| Ile2-Phe10 | -91.41 | 4.28 | 0 |
| Leu13-Ile26 | 113.98 | 4.19 | 0 |
| Ala17-26ile | 76.34 | 4.65 | 0 |
| Thr27-Thr40 | -65.95 | 5.22 | 0 |
| Cys30-Tyr39 | -59.73 | 4.07 | 0 |
| Ser47-Met58 | -62.7 | 4.74 | 0 |
| Thr49-Ile61 | -104.97 | 3.37 | 0 |
| Ala53-Val71 | 100.43 | 6.44 | 0 |
| Phe63-Ala67 | 106.67 | 5.37 | 0 |
| Met89-109 | -80.15 | 0.59 | 0 |
| Ile95-Thr99 | -75.18 | 4.45 | 0 |
| Glu100-Ile120 | 113.9 | 6.54 | 0 |
| Val103-Ala119 | 104.46 | 5.76 | 0 |
| Ala119-Ala132 | 74.01 | 2.77 | 0 |
| Ser121-Pro133 | 111.77 | 2.74 | 0 |
| Met122-Ala126 | 91.55 | 2.7 | 0 |
| Thr134-Ile168 | 95.21 | 1.38 | 0 |
| Ala136-Gly142 | -82.34 | 5.23 | 0 |

**Table S5.** Population coverage analysis of vaccine epitopes.

| population /area | Class I | | | Class II | | | Class combined | | |
|---|---|---|---|---|---|---|---|---|---|
| | coverage[a] | average_hit[b] | pc90[c] | coverage[a] | average_hit[b] | pc90[c] | coverage[a] | average_hit[b] | pc90[c] |
| Algeria | 0.0% | 0.0 | 0.0 | 77.15% | 2.98 | 1.31 | 77.15% | 2.98 | 1.31 |
| | | | | | | | | | |
| Algeria Arab | 0.0% | 0.0 | 0.0 | 77.15% | 2.98 | 1.31 | 77.15% | 2.98 | 1.31 |
| | | | | | | | | | |
| American Samoa | 98.75% | 6.07 | 4.15 | 0.0% | 0.0 | 0.0 | 98.75% | 6.07 | 4.15 |
| | | | | | | | | | |
| American Samoa Polynesian | 98.75% | 6.07 | 4.15 | 0.0% | 0.0 | 0.0 | 98.75% | 6.07 | 4.15 |
| | | | | | | | | | |
| Argentina | 97.5% | 5.3 | 3.66 | 62.67% | 2.27 | 0.8 | 99.07% | 7.58 | 4.96 |
| | | | | | | | | | |
| Argentina Amerindian | 97.5% | 5.3 | 3.66 | 45.78% | 1.52 | 0.55 | 98.64% | 6.82 | 4.34 |
| | | | | | | | | | |
| Argentina Caucasoid | 0.0% | 0.0 | 0.0 | 80.65% | 3.22 | 1.55 | 80.65% | 3.22 | 1.55 |
| | | | | | | | | | |
| Australia | 94.51% | 5.5 | 3.44 | 33.15% | 1.08 | 0.45 | 96.33% | 6.58 | 3.85 |
| | | | | | | | | | |
| Australia Australian Aborigines | 87.31% | 4.07 | 2.36 | 33.15% | 1.08 | 0.45 | 91.52% | 5.14 | 3.13 |
| | | | | | | | | | |
| Australia Caucasoid | 99.94% | 9.13 | 7.07 | 0.0% | 0.0 | 0.0 | 99.94% | 9.13 | 7.07 |
| | | | | | | | | | |
| Austria | 99.91% | 8.6 | 6.6 | 93.34% | 4.21 | 3.22 | 99.99% | 12.81 | 9.98 |
| | | | | | | | | | |
| Austria Caucasoid | 99.91% | 8.6 | 6.6 | 93.34% | 4.21 | 3.22 | 99.99% | 12.81 | 9.98 |
| | | | | | | | | | |
| Belarus | 0.0% | 0.0 | 0.0 | 43.81% | 1.41 | 0.53 | 43.81% | 1.41 | 0.53 |
| | | | | | | | | | |

| | | | | | | | | | |
|---|---|---|---|---|---|---|---|---|---|
| Belarus Caucasoid | 0.0% | 0.0 | 0.0 | 43.81% | 1.41 | 0.53 | 43.81% | 1.41 | 0.53 |
| | | | | | | | | | |
| Belgium | 99.39% | 6.66 | 4.67 | 79.39% | 3.11 | 1.46 | 99.87% | 9.77 | 6.95 |
| | | | | | | | | | |
| Belgium Caucasoid | 99.39% | 6.66 | 4.67 | 79.39% | 3.11 | 1.46 | 99.87% | 9.77 | 6.95 |
| | | | | | | | | | |
| Bolivia | 0.0% | 0.0 | 0.0 | 77.82% | 2.79 | 1.35 | 77.82% | 2.79 | 1.35 |
| | | | | | | | | | |
| Bolivia Amerindian | 0.0% | 0.0 | 0.0 | 77.82% | 2.79 | 1.35 | 77.82% | 2.79 | 1.35 |
| | | | | | | | | | |
| Borneo | 0.0% | 0.0 | 0.0 | 49.02% | 1.62 | 0.59 | 49.02% | 1.62 | 0.59 |
| | | | | | | | | | |
| Borneo Austronesian | 0.0% | 0.0 | 0.0 | 49.02% | 1.62 | 0.59 | 49.02% | 1.62 | 0.59 |
| | | | | | | | | | |
| Brazil | 96.1% | 5.85 | 3.69 | 63.8% | 2.3 | 0.83 | 98.59% | 8.16 | 5.22 |
| | | | | | | | | | |
| Brazil Amerindian | 93.24% | 4.12 | 3.19 | 48.6% | 1.55 | 0.58 | 96.52% | 5.67 | 3.66 |
| | | | | | | | | | |
| Brazil Caucasoid | 99.32% | 7.37 | 5.29 | 84.39% | 3.49 | 1.92 | 99.89% | 10.86 | 7.82 |
| | | | | | | | | | |
| Brazil Mixed | 97.02% | 6.41 | 3.99 | 77.5% | 3.03 | 1.33 | 99.33% | 9.44 | 6.41 |
| | | | | | | | | | |
| Brazil Mulatto | 0.0% | 0.0 | 0.0 | 74.09% | 2.85 | 1.16 | 74.09% | 2.85 | 1.16 |
| | | | | | | | | | |
| Bulgaria | 99.42% | 7.1 | 5.1 | 57.23% | 2.01 | 0.7 | 99.75% | 9.11 | 6.46 |
| | | | | | | | | | |
| Bulgaria Caucasoid | 99.52% | 7.2 | 5.26 | 57.23% | 2.01 | 0.7 | 99.79% | 9.21 | 6.53 |
| | | | | | | | | | |
| Bulgaria Other | 99.58% | 6.19 | 4.31 | 0.0% | 0.0 | 0.0 | 99.58% | 6.19 | 4.31 |

| | | | | | | | | | |
|---|---|---|---|---|---|---|---|---|---|
| Burkina Faso | 67.18% | 2.77 | 0.91 | 0.0% | 0.0 | 0.0 | 67.18% | 2.77 | 0.91 |
| | | | | | | | | | |
| Burkina Faso Black | 67.18% | 2.77 | 0.91 | 0.0% | 0.0 | 0.0 | 67.18% | 2.77 | 0.91 |
| | | | | | | | | | |
| Cameroon | 89.72% | 5.0 | 2.92 | 49.87% | 1.7 | 0.6 | 94.85% | 6.7 | 3.68 |
| | | | | | | | | | |
| Cameroon Black | 89.72% | 5.0 | 2.92 | 49.87% | 1.7 | 0.6 | 94.85% | 6.7 | 3.68 |
| | | | | | | | | | |
| Canada | 0.0% | 0.0 | 0.0 | 38.41% | 1.26 | 0.49 | 38.41% | 1.26 | 0.49 |
| | | | | | | | | | |
| Canada Amerindian | 0.0% | 0.0 | 0.0 | 38.41% | 1.26 | 0.49 | 38.41% | 1.26 | 0.49 |
| | | | | | | | | | |
| Cape Verde | 99.18% | 7.76 | 5.74 | 80.38% | 3.23 | 1.53 | 99.84% | 10.99 | 7.84 |
| | | | | | | | | | |
| Cape Verde Black | 99.18% | 7.76 | 5.74 | 80.38% | 3.23 | 1.53 | 99.84% | 10.99 | 7.84 |
| | | | | | | | | | |
| Central Africa | 86.04% | 4.5 | 2.15 | 62.71% | 2.26 | 0.8 | 94.79% | 6.76 | 3.68 |
| | | | | | | | | | |
| Central African Republic | 28.02% | 0.91 | 0.42 | 82.69% | 3.21 | 1.73 | 87.54% | 4.12 | 2.41 |
| | | | | | | | | | |
| Central African Republic Black | 28.02% | 0.91 | 0.42 | 82.69% | 3.21 | 1.73 | 87.54% | 4.12 | 2.41 |
| | | | | | | | | | |
| Central America | 7.76% | 0.24 | 0.33 | 49.91% | 1.68 | 0.6 | 53.8% | 1.92 | 0.65 |
| | | | | | | | | | |
| Chile | 95.94% | 5.73 | 3.63 | 67.08% | 2.5 | 0.91 | 98.66% | 8.22 | 5.3 |
| | | | | | | | | | |
| Chile Amerindian | 99.63% | 6.56 | 4.49 | 72.65% | 2.63 | 1.1 | 99.9% | 9.2 | 6.56 |
| | | | | | | | | | |

| | | | | | | | | | |
|---|---|---|---|---|---|---|---|---|---|
| Chile Mixed | 90.09% | 4.82 | 3.01 | 52.65% | 1.83 | 0.63 | 95.31% | 6.64 | 3.73 |
| | | | | | | | | | |
| China | 94.57% | 5.41 | 3.43 | 59.99% | 2.15 | 0.75 | 97.83% | 7.57 | 4.53 |
| | | | | | | | | | |
| China Oriental | 94.57% | 5.41 | 3.43 | 59.99% | 2.15 | 0.75 | 97.83% | 7.57 | 4.53 |
| | | | | | | | | | |
| Colombia | 8.36% | 0.25 | 0.33 | 54.02% | 1.88 | 0.65 | 57.86% | 2.13 | 0.71 |
| | | | | | | | | | |
| Colombia Amerindian | 0.0% | 0.0 | 0.0 | 47.4% | 1.59 | 0.57 | 47.4% | 1.59 | 0.57 |
| | | | | | | | | | |
| Colombia Black | 3.65% | 0.11 | 0.31 | 65.25% | 2.4 | 0.86 | 66.51% | 2.51 | 0.9 |
| | | | | | | | | | |
| Colombia Mestizo | 14.07% | 0.42 | 0.35 | 56.31% | 1.97 | 0.69 | 62.45% | 2.39 | 0.8 |
| | | | | | | | | | |
| Congo | 0.0% | 0.0 | 0.0 | 68.66% | 2.54 | 0.96 | 68.66% | 2.54 | 0.96 |
| | | | | | | | | | |
| Congo Black | 0.0% | 0.0 | 0.0 | 68.66% | 2.54 | 0.96 | 68.66% | 2.54 | 0.96 |
| | | | | | | | | | |
| Cook Islands | 0.0% | 0.0 | 0.0 | 78.59% | 3.05 | 1.4 | 78.59% | 3.05 | 1.4 |
| | | | | | | | | | |
| Cook Islands Polynesian | 0.0% | 0.0 | 0.0 | 78.59% | 3.05 | 1.4 | 78.59% | 3.05 | 1.4 |
| | | | | | | | | | |
| Costa Rica | 0.0% | 0.0 | 0.0 | 24.31% | 0.76 | 0.4 | 24.31% | 0.76 | 0.4 |
| | | | | | | | | | |
| Costa Rica Mestizo | 0.0% | 0.0 | 0.0 | 24.31% | 0.76 | 0.4 | 24.31% | 0.76 | 0.4 |
| | | | | | | | | | |
| Croatia | 99.79% | 7.96 | 6.18 | 66.71% | 2.45 | 0.9 | 99.93% | 10.4 | 7.43 |
| | | | | | | | | | |
| Croatia Caucasoid | 99.79% | 7.96 | 6.18 | 66.71% | 2.45 | 0.9 | 99.93% | 10.4 | 7.43 |

| | | | | | | | | | |
|---|---|---|---|---|---|---|---|---|---|
| Cuba | 98.92% | 7.47 | 5.19 | 85.48% | 3.44 | 2.07 | 99.84% | 10.91 | 7.81 |
| | | | | | | | | | |
| Cuba Caucasoid | 99.05% | 7.42 | 5.21 | 0.0% | 0.0 | 0.0 | 99.05% | 7.42 | 5.21 |
| | | | | | | | | | |
| Cuba Mixed | 0.0% | 0.0 | 0.0 | 85.48% | 3.44 | 2.07 | 85.48% | 3.44 | 2.07 |
| | | | | | | | | | |
| Cuba Mulatto | 98.74% | 7.48 | 5.13 | 0.0% | 0.0 | 0.0 | 98.74% | 7.48 | 5.13 |
| | | | | | | | | | |
| Czech Republic | 99.08% | 7.49 | 5.27 | 86.21% | 3.58 | 2.18 | 99.87% | 11.07 | 7.99 |
| | | | | | | | | | |
| Czech Republic Caucasoid | 99.08% | 7.49 | 5.27 | 88.76% | 3.78 | 2.67 | 99.9% | 11.27 | 8.29 |
| | | | | | | | | | |
| Czech Republic Other | 0.0% | 0.0 | 0.0 | 64.14% | 2.29 | 0.84 | 64.14% | 2.29 | 0.84 |
| | | | | | | | | | |
| Denmark | 0.0% | 0.0 | 0.0 | 88.98% | 3.73 | 2.72 | 88.98% | 3.73 | 2.72 |
| | | | | | | | | | |
| Denmark Caucasoid | 0.0% | 0.0 | 0.0 | 88.98% | 3.73 | 2.72 | 88.98% | 3.73 | 2.72 |
| | | | | | | | | | |
| East Africa | 90.78% | 5.03 | 3.07 | 68.3% | 2.51 | 0.95 | 97.08% | 7.54 | 4.35 |
| | | | | | | | | | |
| East Asia | 98.18% | 6.69 | 4.31 | 81.82% | 3.32 | 1.65 | 99.67% | 10.01 | 6.89 |
| | | | | | | | | | |
| Ecuador | 77.35% | 2.65 | 1.32 | 52.17% | 1.69 | 0.63 | 89.16% | 4.34 | 2.77 |
| | | | | | | | | | |
| Ecuador Amerindian | 77.35% | 2.65 | 1.32 | 52.17% | 1.69 | 0.63 | 89.16% | 4.34 | 2.77 |
| | | | | | | | | | |
| England | 99.93% | 9.02 | 6.95 | 93.48% | 4.21 | 3.22 | 100.0% | 13.24 | 10.42 |
| | | | | | | | | | |

| | | | | | | | | | |
|---|---|---|---|---|---|---|---|---|---|
| England Caucasoid | 99.93% | 9.02 | 6.95 | 93.48% | 4.21 | 3.22 | 100.0% | 13.24 | 10.42 |
| | | | | | | | | | |
| Equatorial Guinea | 0.0% | 0.0 | 0.0 | 47.58% | 1.61 | 0.57 | 47.58% | 1.61 | 0.57 |
| | | | | | | | | | |
| Equatorial Guinea Black | 0.0% | 0.0 | 0.0 | 47.58% | 1.61 | 0.57 | 47.58% | 1.61 | 0.57 |
| | | | | | | | | | |
| Ethiopia | 0.0% | 0.0 | 0.0 | 83.0% | 3.27 | 1.76 | 83.0% | 3.27 | 1.76 |
| | | | | | | | | | |
| Ethiopia Black | 0.0% | 0.0 | 0.0 | 83.0% | 3.27 | 1.76 | 83.0% | 3.27 | 1.76 |
| | | | | | | | | | |
| Europe | 99.68% | 8.05 | 6.17 | 85.83% | 3.57 | 2.12 | 99.96% | 11.62 | 8.97 |
| | | | | | | | | | |
| Fiji | 0.0% | 0.0 | 0.0 | 79.87% | 2.95 | 1.49 | 79.87% | 2.95 | 1.49 |
| | | | | | | | | | |
| Fiji Melanesian | 0.0% | 0.0 | 0.0 | 79.87% | 2.95 | 1.49 | 79.87% | 2.95 | 1.49 |
| | | | | | | | | | |
| Finland | 99.99% | 9.19 | 7.16 | 51.14% | 1.75 | 0.61 | 100.0% | 10.94 | 8.49 |
| | | | | | | | | | |
| Finland Caucasoid | 99.99% | 9.19 | 7.16 | 51.14% | 1.75 | 0.61 | 100.0% | 10.94 | 8.49 |
| | | | | | | | | | |
| France | 99.8% | 8.26 | 6.34 | 88.54% | 3.78 | 2.62 | 99.98% | 12.04 | 9.31 |
| | | | | | | | | | |
| France Caucasoid | 99.8% | 8.26 | 6.34 | 88.54% | 3.78 | 2.62 | 99.98% | 12.04 | 9.31 |
| | | | | | | | | | |
| Gabon | 0.0% | 0.0 | 0.0 | 41.78% | 1.35 | 0.52 | 41.78% | 1.35 | 0.52 |
| | | | | | | | | | |
| Gabon Black | 0.0% | 0.0 | 0.0 | 41.78% | 1.35 | 0.52 | 41.78% | 1.35 | 0.52 |
| | | | | | | | | | |
| Georgia | 98.32% | 6.77 | 4.41 | 75.05% | 2.85 | 1.2 | 99.58% | 9.62 | 6.63 |

| | | | | | | | | | |
|---|---|---|---|---|---|---|---|---|---|
| Georgia Caucasoid | 98.94% | 7.1 | 4.83 | 75.05% | 2.85 | 1.2 | 99.74% | 9.95 | 6.91 |
| | | | | | | | | | |
| Georgia Kurd | 98.19% | 6.69 | 4.37 | 0.0% | 0.0 | 0.0 | 98.19% | 6.69 | 4.37 |
| | | | | | | | | | |
| Germany | 99.93% | 8.86 | 6.8 | 91.14% | 4.0 | 3.07 | 99.99% | 12.86 | 10.0 |
| | | | | | | | | | |
| Germany Caucasoid | 99.93% | 8.86 | 6.8 | 91.14% | 4.0 | 3.07 | 99.99% | 12.86 | 10.0 |
| | | | | | | | | | |
| Greece | 0.0% | 0.0 | 0.0 | 66.92% | 2.47 | 0.91 | 66.92% | 2.47 | 0.91 |
| | | | | | | | | | |
| Greece Caucasoid | 0.0% | 0.0 | 0.0 | 66.92% | 2.47 | 0.91 | 66.92% | 2.47 | 0.91 |
| | | | | | | | | | |
| Guatemala | 7.76% | 0.24 | 0.33 | 49.16% | 1.61 | 0.59 | 53.11% | 1.85 | 0.64 |
| | | | | | | | | | |
| Guatemala Amerindian | 7.76% | 0.24 | 0.33 | 49.16% | 1.61 | 0.59 | 53.11% | 1.85 | 0.64 |
| | | | | | | | | | |
| Guinea-Bissau | 96.39% | 6.37 | 3.89 | 71.16% | 2.67 | 1.04 | 98.96% | 9.04 | 6.11 |
| | | | | | | | | | |
| Guinea-Bissau Black | 96.39% | 6.37 | 3.89 | 71.16% | 2.67 | 1.04 | 98.96% | 9.04 | 6.11 |
| | | | | | | | | | |
| Hong Kong | 96.05% | 5.73 | 3.64 | 0.0% | 0.0 | 0.0 | 96.05% | 5.73 | 3.64 |
| | | | | | | | | | |
| Hong Kong Oriental | 96.05% | 5.73 | 3.64 | 0.0% | 0.0 | 0.0 | 96.05% | 5.73 | 3.64 |
| | | | | | | | | | |
| India | 89.41% | 4.74 | 2.83 | 74.99% | 2.87 | 1.2 | 97.35% | 7.6 | 4.46 |
| | | | | | | | | | |
| India Asian | 89.41% | 4.74 | 2.83 | 74.99% | 2.87 | 1.2 | 97.35% | 7.6 | 4.46 |
| | | | | | | | | | |
| Indonesia | 86.79% | 4.38 | 2.27 | 47.84% | 1.62 | 0.58 | 93.11% | 6.0 | 3.35 |

| | | | | | | | | | |
|---|---|---|---|---|---|---|---|---|---|
| Indonesia Austronesian | 86.79% | 4.38 | 2.27 | 47.84% | 1.62 | 0.58 | 93.11% | 6.0 | 3.35 |
| | | | | | | | | | |
| Iran | 97.5% | 6.56 | 4.14 | 64.22% | 2.32 | 0.84 | 99.11% | 8.89 | 6.09 |
| | | | | | | | | | |
| Iran Kurd | 0.0% | 0.0 | 0.0 | 55.78% | 1.94 | 0.68 | 55.78% | 1.94 | 0.68 |
| | | | | | | | | | |
| Iran Persian | 97.5% | 6.56 | 4.14 | 65.72% | 2.39 | 0.88 | 99.14% | 8.96 | 6.13 |
| | | | | | | | | | |
| Ireland Northern | 99.94% | 9.18 | 7.11 | 94.65% | 4.3 | 3.3 | 100.0% | 13.48 | 10.76 |
| | | | | | | | | | |
| Ireland Northern Caucasoid | 99.94% | 9.18 | 7.11 | 94.65% | 4.3 | 3.3 | 100.0% | 13.48 | 10.76 |
| | | | | | | | | | |
| Ireland South | 99.93% | 9.02 | 6.96 | 93.15% | 4.12 | 3.19 | 100.0% | 13.15 | 10.35 |
| | | | | | | | | | |
| Ireland South Caucasoid | 99.93% | 9.02 | 6.96 | 93.15% | 4.12 | 3.19 | 100.0% | 13.15 | 10.35 |
| | | | | | | | | | |
| Israel | 89.39% | 4.68 | 2.83 | 68.79% | 2.53 | 0.96 | 96.69% | 7.21 | 4.14 |
| | | | | | | | | | |
| Israel Arab | 94.42% | 5.58 | 3.45 | 67.51% | 2.48 | 0.92 | 98.19% | 8.06 | 4.96 |
| | | | | | | | | | |
| Israel Jew | 93.75% | 5.21 | 3.33 | 69.65% | 2.57 | 0.99 | 98.1% | 7.78 | 4.79 |
| | | | | | | | | | |
| Italy | 99.03% | 7.54 | 5.34 | 85.9% | 3.28 | 2.13 | 99.86% | 10.82 | 7.84 |
| | | | | | | | | | |
| Italy Caucasoid | 99.03% | 7.54 | 5.34 | 85.9% | 3.28 | 2.13 | 99.86% | 10.82 | 7.84 |
| | | | | | | | | | |
| Ivory Coast | 67.75% | 2.41 | 0.93 | 0.0% | 0.0 | 0.0 | 67.75% | 2.41 | 0.93 |
| | | | | | | | | | |

| | | | | | | | | | |
|---|---|---|---|---|---|---|---|---|---|
| Ivory Coast Black | 67.75 % | 2.41 | 0.93 | 0.0% | 0.0 | 0.0 | 67.75 % | 2.41 | 0.93 |
| | | | | | | | | | |
| Jamaica | 0.0% | 0.0 | 0.0 | 27.41 % | 0.87 | 0.41 | 27.41 % | 0.87 | 0.41 |
| | | | | | | | | | |
| Jamaica Black | 0.0% | 0.0 | 0.0 | 27.41 % | 0.87 | 0.41 | 27.41 % | 0.87 | 0.41 |
| | | | | | | | | | |
| Japan | 98.63 % | 6.84 | 4.5 | 74.83 % | 2.87 | 1.19 | 99.66 % | 9.72 | 6.69 |
| | | | | | | | | | |
| Japan Oriental | 98.63 % | 6.84 | 4.5 | 74.83 % | 2.87 | 1.19 | 99.66 % | 9.72 | 6.69 |
| | | | | | | | | | |
| Jordan | 90.83 % | 4.71 | 3.06 | 52.88 % | 1.8 | 0.64 | 95.68 % | 6.52 | 3.76 |
| | | | | | | | | | |
| Jordan Arab | 90.83 % | 4.71 | 3.06 | 52.88 % | 1.8 | 0.64 | 95.68 % | 6.52 | 3.76 |
| | | | | | | | | | |
| Kenya | 89.56 % | 4.84 | 2.87 | 0.0% | 0.0 | 0.0 | 89.56 % | 4.84 | 2.87 |
| | | | | | | | | | |
| Kenya Black | 89.56 % | 4.84 | 2.87 | 0.0% | 0.0 | 0.0 | 89.56 % | 4.84 | 2.87 |
| | | | | | | | | | |
| Kiribati | 0.0% | 0.0 | 0.0 | 10.89 % | 0.33 | 0.34 | 10.89 % | 0.33 | 0.34 |
| | | | | | | | | | |
| Kiribati Micronesian | 0.0% | 0.0 | 0.0 | 10.89 % | 0.33 | 0.34 | 10.89 % | 0.33 | 0.34 |
| | | | | | | | | | |
| Korea; South | 98.21 % | 6.87 | 4.44 | 85.32 % | 3.57 | 2.04 | 99.74 % | 10.44 | 7.24 |
| | | | | | | | | | |
| Korea; South Oriental | 98.21 % | 6.87 | 4.44 | 85.32 % | 3.57 | 2.04 | 99.74 % | 10.44 | 7.24 |
| | | | | | | | | | |
| Lebanon | 0.0% | 0.0 | 0.0 | 70.46 % | 2.62 | 1.02 | 70.46 % | 2.62 | 1.02 |
| | | | | | | | | | |

| | | | | | | | | | |
|---|---|---|---|---|---|---|---|---|---|
| Lebanon Arab | 0.0% | 0.0 | 0.0 | 70.46% | 2.62 | 1.02 | 70.46% | 2.62 | 1.02 |
| | | | | | | | | | |
| Macedonia | 26.72% | 0.87 | 0.41 | 66.53% | 2.45 | 0.9 | 75.47% | 3.31 | 1.22 |
| | | | | | | | | | |
| Macedonia Caucasoid | 26.72% | 0.87 | 0.41 | 66.53% | 2.45 | 0.9 | 75.47% | 3.31 | 1.22 |
| | | | | | | | | | |
| Malaysia | 81.38% | 3.71 | 1.61 | 57.99% | 2.05 | 0.71 | 92.18% | 5.76 | 3.23 |
| | | | | | | | | | |
| Malaysia Austronesian | 63.18% | 2.52 | 0.81 | 55.38% | 1.94 | 0.67 | 83.57% | 4.46 | 1.83 |
| | | | | | | | | | |
| Malaysia Oriental | 87.82% | 4.26 | 2.46 | 70.35% | 2.58 | 1.01 | 96.39% | 6.84 | 3.97 |
| | | | | | | | | | |
| Mali | 96.02% | 6.04 | 3.73 | 0.0% | 0.0 | 0.0 | 96.02% | 6.04 | 3.73 |
| | | | | | | | | | |
| Mali Black | 96.02% | 6.04 | 3.73 | 0.0% | 0.0 | 0.0 | 96.02% | 6.04 | 3.73 |
| | | | | | | | | | |
| Martinique | 22.56% | 0.68 | 0.39 | 74.51% | 2.88 | 1.18 | 80.26% | 3.56 | 1.52 |
| | | | | | | | | | |
| Martinique Black | 22.56% | 0.68 | 0.39 | 74.51% | 2.88 | 1.18 | 80.26% | 3.56 | 1.52 |
| | | | | | | | | | |
| Mexico | 97.97% | 5.98 | 3.95 | 55.04% | 1.91 | 0.67 | 99.09% | 7.89 | 5.23 |
| | | | | | | | | | |
| Mexico Amerindian | 99.87% | 6.68 | 4.95 | 42.59% | 1.38 | 0.52 | 99.93% | 8.06 | 6.1 |
| | | | | | | | | | |
| Mexico Mestizo | 98.13% | 6.24 | 4.09 | 68.51% | 2.54 | 0.95 | 99.41% | 8.77 | 6.16 |
| | | | | | | | | | |
| Mongolia | 95.31% | 4.43 | 3.37 | 81.85% | 3.34 | 1.65 | 99.15% | 7.77 | 5.67 |
| | | | | | | | | | |
| Mongolia Oriental | 95.31% | 4.43 | 3.37 | 81.85% | 3.34 | 1.65 | 99.15% | 7.77 | 5.67 |

| | | | | | | | | | |
|---|---|---|---|---|---|---|---|---|---|
| Morocco | 98.63 % | 6.97 | 4.6 6 | 83.44 % | 3.36 | 1.8 1 | 99.77 % | 10.33 | 7.2 3 |
| | | | | | | | | | |
| Morocco Arab | 99.32 % | 7.46 | 5.4 7 | 85.07 % | 3.47 | 2.0 1 | 99.9% | 10.93 | 7.9 7 |
| | | | | | | | | | |
| Morocco Caucasoid | 97.94 % | 6.6 | 4.2 5 | 79.75 % | 3.11 | 1.4 8 | 99.58 % | 9.72 | 6.7 |
| | | | | | | | | | |
| Nauru | 0.0% | 0.0 | 0.0 | 38.66 % | 1.26 | 0.4 9 | 38.66 % | 1.26 | 0.4 9 |
| | | | | | | | | | |
| Nauru Micronesia n | 0.0% | 0.0 | 0.0 | 38.66 % | 1.26 | 0.4 9 | 38.66 % | 1.26 | 0.4 9 |
| | | | | | | | | | |
| Netherland s | 0.0% | 0.0 | 0.0 | 83.44 % | 3.41 | 1.8 1 | 83.44 % | 3.41 | 1.8 1 |
| | | | | | | | | | |
| Netherland s Caucasoid | 0.0% | 0.0 | 0.0 | 83.44 % | 3.41 | 1.8 1 | 83.44 % | 3.41 | 1.8 1 |
| | | | | | | | | | |
| New Caledonia | 98.18 % | 4.94 | 3.5 5 | 81.41 % | 3.02 | 1.6 1 | 99.66 % | 7.96 | 6.0 2 |
| | | | | | | | | | |
| New Caledonia Melanesian | 98.18 % | 4.94 | 3.5 5 | 81.41 % | 3.02 | 1.6 1 | 99.66 % | 7.96 | 6.0 2 |
| | | | | | | | | | |
| New Zealand | 0.0% | 0.0 | 0.0 | 84.46 % | 3.42 | 1.9 3 | 84.46 % | 3.42 | 1.9 3 |
| | | | | | | | | | |
| New Zealand Polynesian | 0.0% | 0.0 | 0.0 | 84.46 % | 3.42 | 1.9 3 | 84.46 % | 3.42 | 1.9 3 |
| | | | | | | | | | |
| Niue | 0.0% | 0.0 | 0.0 | 77.82 % | 2.86 | 1.3 5 | 77.82 % | 2.86 | 1.3 5 |
| | | | | | | | | | |
| Niue Polynesian | 0.0% | 0.0 | 0.0 | 77.82 % | 2.86 | 1.3 5 | 77.82 % | 2.86 | 1.3 5 |
| | | | | | | | | | |
| North Africa | 96.03 % | 5.98 | 3.7 2 | 75.06 % | 2.87 | 1.2 | 99.01 % | 8.85 | 6.0 7 |
| | | | | | | | | | |

| | | | | | | | | | |
|---|---|---|---|---|---|---|---|---|---|
| North America | 99.06% | 7.34 | 5.1 | 87.89% | 3.74 | 2.48 | 99.89% | 11.09 | 8.04 |
| | | | | | | | | | |
| Northeast Asia | 94.7% | 5.45 | 3.45 | 59.99% | 2.15 | 0.75 | 97.88% | 7.6 | 4.56 |
| | | | | | | | | | |
| Norway | 0.0% | 0.0 | 0.0 | 94.71% | 4.34 | 3.32 | 94.71% | 4.34 | 3.32 |
| | | | | | | | | | |
| Norway Caucasoid | 0.0% | 0.0 | 0.0 | 94.71% | 4.34 | 3.32 | 94.71% | 4.34 | 3.32 |
| | | | | | | | | | |
| Oceania | 94.71% | 4.99 | 3.37 | 59.87% | 2.13 | 0.75 | 97.88% | 7.12 | 4.31 |
| | | | | | | | | | |
| Oman | 99.69% | 7.84 | 6.13 | 0.0% | 0.0 | 0.0 | 99.69% | 7.84 | 6.13 |
| | | | | | | | | | |
| Oman Arab | 99.69% | 7.84 | 6.13 | 0.0% | 0.0 | 0.0 | 99.69% | 7.84 | 6.13 |
| | | | | | | | | | |
| Pakistan | 97.09% | 4.77 | 3.61 | 1.18% | 0.04 | 0.3 | 97.13% | 4.81 | 3.62 |
| | | | | | | | | | |
| Pakistan Asian | 96.75% | 4.73 | 3.56 | 1.45% | 0.04 | 0.3 | 96.79% | 4.77 | 3.58 |
| | | | | | | | | | |
| Pakistan Mixed | 97.73% | 4.84 | 3.68 | 0.0% | 0.0 | 0.3 | 97.73% | 4.84 | 3.68 |
| | | | | | | | | | |
| Papua New Guinea | 97.92% | 4.45 | 3.45 | 69.15% | 2.52 | 0.97 | 99.36% | 6.97 | 4.62 |
| | | | | | | | | | |
| Papua New Guinea Melanesian | 97.92% | 4.45 | 3.45 | 69.15% | 2.52 | 0.97 | 99.36% | 6.97 | 4.62 |
| | | | | | | | | | |
| Paraguay | 0.0% | 0.0 | 0.0 | 4.9% | 0.15 | 0.32 | 4.9% | 0.15 | 0.32 |
| | | | | | | | | | |
| Paraguay Amerindian | 0.0% | 0.0 | 0.0 | 4.9% | 0.15 | 0.32 | 4.9% | 0.15 | 0.32 |
| | | | | | | | | | |
| Peru | 99.99% | 5.89 | 4.19 | 49.87% | 1.64 | 0.6 | 100.0% | 7.54 | 5.36 |

| | | | | | | | | | |
|---|---|---|---|---|---|---|---|---|---|
| Peru Amerindian | 99.99% | 5.89 | 4.19 | 49.87% | 1.64 | 0.6 | 100.0% | 7.54 | 5.36 |
| | | | | | | | | | |
| Philippines | 94.98% | 4.72 | 3.35 | 28.56% | 0.91 | 0.42 | 96.41% | 5.63 | 3.6 |
| | | | | | | | | | |
| Philippines Austronesian | 94.98% | 4.72 | 3.35 | 28.56% | 0.91 | 0.42 | 96.41% | 5.63 | 3.6 |
| | | | | | | | | | |
| Poland | 99.77% | 8.06 | 6.21 | 84.46% | 3.47 | 1.93 | 99.96% | 11.53 | 8.87 |
| | | | | | | | | | |
| Poland Caucasoid | 99.77% | 8.06 | 6.21 | 84.46% | 3.47 | 1.93 | 99.96% | 11.53 | 8.87 |
| | | | | | | | | | |
| Portugal | 98.72% | 7.11 | 4.76 | 78.0% | 3.05 | 1.36 | 99.72% | 10.15 | 7.02 |
| | | | | | | | | | |
| Portugal Caucasoid | 98.72% | 7.11 | 4.76 | 78.0% | 3.05 | 1.36 | 99.72% | 10.15 | 7.02 |
| | | | | | | | | | |
| Romania | 99.67% | 7.7 | 6.02 | 0.0% | 0.0 | 0.0 | 99.67% | 7.7 | 6.02 |
| | | | | | | | | | |
| Romania Caucasoid | 99.67% | 7.7 | 6.02 | 0.0% | 0.0 | 0.0 | 99.67% | 7.7 | 6.02 |
| | | | | | | | | | |
| Russia | 99.27% | 7.18 | 5.07 | 77.62% | 3.07 | 1.34 | 99.84% | 10.25 | 7.18 |
| | | | | | | | | | |
| Russia Caucasoid | 3.96% | 0.12 | 0.31 | 88.52% | 3.79 | 2.61 | 88.97% | 3.91 | 2.72 |
| | | | | | | | | | |
| Russia Mixed | 5.05% | 0.15 | 0.32 | 0.0% | 0.0 | 0.0 | 5.05% | 0.15 | 0.32 |
| | | | | | | | | | |
| Russia Other | 99.98% | 7.54 | 6.15 | 85.01% | 3.48 | 2.0 | 100.0% | 11.02 | 8.5 |
| | | | | | | | | | |
| Russia Siberian | 99.43% | 7.64 | 5.7 | 78.83% | 3.13 | 1.42 | 99.88% | 10.77 | 7.66 |
| | | | | | | | | | |
| Rwanda | 24.87% | 0.8 | 0.4 | 62.79% | 2.24 | 0.81 | 72.05% | 3.04 | 1.07 |

| | | | | | | | | | |
|---|---|---|---|---|---|---|---|---|---|
| Rwanda Black | 24.87 % | 0.8 | 0.4 | 62.79 % | 2.24 | 0.8 1 | 72.05 % | 3.04 | 1.0 7 |
| | | | | | | | | | |
| Samoa | 0.0% | 0.0 | 0.0 | 80.86 % | 3.18 | 1.5 7 | 80.86 % | 3.18 | 1.5 7 |
| | | | | | | | | | |
| Samoa Polynesian | 0.0% | 0.0 | 0.0 | 80.86 % | 3.18 | 1.5 7 | 80.86 % | 3.18 | 1.5 7 |
| | | | | | | | | | |
| Sao Tome and Principe | 97.02 % | 6.65 | 4.1 2 | 66.5% | 2.43 | 0.9 | 99.0% | 9.08 | 6.1 5 |
| | | | | | | | | | |
| Sao Tome and Principe Black | 97.02 % | 6.65 | 4.1 2 | 66.5% | 2.43 | 0.9 | 99.0% | 9.08 | 6.1 5 |
| | | | | | | | | | |
| Saudi Arabia | 98.26 % | 6.61 | 4.3 4 | 80.14 % | 3.15 | 1.5 1 | 99.65 % | 9.75 | 6.7 8 |
| | | | | | | | | | |
| Saudi Arabia Arab | 98.26 % | 6.61 | 4.3 4 | 80.14 % | 3.15 | 1.5 1 | 99.65 % | 9.75 | 6.7 8 |
| | | | | | | | | | |
| Scotland | 65.34 % | 2.62 | 0.8 7 | 90.82 % | 3.94 | 3.0 5 | 96.82 % | 6.56 | 3.9 5 |
| | | | | | | | | | |
| Scotland Caucasoid | 65.34 % | 2.62 | 0.8 7 | 90.82 % | 3.94 | 3.0 5 | 96.82 % | 6.56 | 3.9 5 |
| | | | | | | | | | |
| Senegal | 95.58 % | 5.94 | 3.6 5 | 30.28 % | 0.98 | 0.4 3 | 96.92 % | 6.91 | 4.0 8 |
| | | | | | | | | | |
| Senegal Black | 95.58 % | 5.94 | 3.6 5 | 30.28 % | 0.98 | 0.4 3 | 96.92 % | 6.91 | 4.0 8 |
| | | | | | | | | | |
| Serbia | 73.37 % | 2.8 | 1.1 3 | 0.0% | 0.0 | 0.0 | 73.37 % | 2.8 | 1.1 3 |
| | | | | | | | | | |
| Serbia Caucasoid | 73.37 % | 2.8 | 1.1 3 | 0.0% | 0.0 | 0.0 | 73.37 % | 2.8 | 1.1 3 |
| | | | | | | | | | |
| Singapore | 92.66 % | 5.13 | 3.2 3 | 65.78 % | 2.4 | 0.8 8 | 97.49 % | 7.53 | 4.4 4 |

| | | | | | | | | | |
|---|---|---|---|---|---|---|---|---|---|
| Singapore Austronesian | 90.55% | 4.86 | 3.05 | 65.78% | 2.4 | 0.88 | 96.77% | 7.26 | 4.17 |
| | | | | | | | | | |
| Singapore Oriental | 94.81% | 5.51 | 3.47 | 0.0% | 0.0 | 0.0 | 94.81% | 5.51 | 3.47 |
| | | | | | | | | | |
| Slovakia | 0.0% | 0.0 | 0.0 | 18.28% | 0.55 | 0.37 | 18.28% | 0.55 | 0.37 |
| | | | | | | | | | |
| Slovakia Caucasoid | 0.0% | 0.0 | 0.0 | 18.28% | 0.55 | 0.37 | 18.28% | 0.55 | 0.37 |
| | | | | | | | | | |
| Slovenia | 0.0% | 0.0 | 0.0 | 84.85% | 3.5 | 1.98 | 84.85% | 3.5 | 1.98 |
| | | | | | | | | | |
| Slovenia Caucasoid | 0.0% | 0.0 | 0.0 | 84.85% | 3.5 | 1.98 | 84.85% | 3.5 | 1.98 |
| | | | | | | | | | |
| South Africa | 93.03% | 5.35 | 3.29 | 32.1% | 1.0 | 0.44 | 95.27% | 6.35 | 3.67 |
| | | | | | | | | | |
| South Africa Black | 91.96% | 5.24 | 3.18 | 32.1% | 1.0 | 0.44 | 94.54% | 6.23 | 3.56 |
| | | | | | | | | | |
| South Africa Other | 97.61% | 6.31 | 4.03 | 0.0% | 0.0 | 0.0 | 97.61% | 6.31 | 4.03 |
| | | | | | | | | | |
| South America | 88.3% | 4.38 | 2.56 | 58.59% | 2.08 | 0.72 | 95.15% | 6.46 | 3.68 |
| | | | | | | | | | |
| South Asia | 94.73% | 5.46 | 3.46 | 75.38% | 2.89 | 1.22 | 98.7% | 8.34 | 5.51 |
| | | | | | | | | | |
| Southeast Asia | 94.56% | 5.41 | 3.43 | 56.98% | 2.01 | 0.7 | 97.66% | 7.42 | 4.41 |
| | | | | | | | | | |
| Southwest Asia | 92.5% | 5.18 | 3.22 | 43.93% | 1.48 | 0.54 | 95.79% | 6.66 | 3.81 |
| | | | | | | | | | |
| Spain | 87.52% | 4.55 | 2.4 | 80.51% | 3.19 | 1.54 | 97.57% | 7.74 | 4.6 |
| | | | | | | | | | |

| | | | | | | | | | |
|---|---|---|---|---|---|---|---|---|---|
| Spain Caucasoid | 87.52% | 4.55 | 2.4 | 80.84% | 3.21 | 1.57 | 97.61% | 7.76 | 4.62 |
| | | | | | | | | | |
| Spain Other | 0.0% | 0.0 | 0.0 | 6.3% | 0.19 | 0.32 | 6.3% | 0.19 | 0.32 |
| | | | | | | | | | |
| Sri Lanka | 52.39% | 1.78 | 0.63 | 0.0% | 0.0 | 0.0 | 52.39% | 1.78 | 0.63 |
| | | | | | | | | | |
| Sri Lanka Asian | 52.39% | 1.78 | 0.63 | 0.0% | 0.0 | 0.0 | 52.39% | 1.78 | 0.63 |
| | | | | | | | | | |
| Sudan | 93.78% | 5.34 | 3.36 | 60.56% | 2.13 | 0.76 | 97.55% | 7.47 | 4.45 |
| | | | | | | | | | |
| Sudan Arab | 70.21% | 2.77 | 1.01 | 0.0% | 0.0 | 0.0 | 70.21% | 2.77 | 1.01 |
| | | | | | | | | | |
| Sudan Black | 2.19% | 0.07 | 0.31 | 0.0% | 0.0 | 0.0 | 2.19% | 0.07 | 0.31 |
| | | | | | | | | | |
| Sudan Mixed | 94.39% | 5.53 | 3.44 | 60.56% | 2.13 | 0.76 | 97.79% | 7.66 | 4.6 |
| | | | | | | | | | |
| Sweden | 99.99% | 9.0 | 7.0 | 88.07% | 3.76 | 2.51 | 100.0% | 12.77 | 9.96 |
| | | | | | | | | | |
| Sweden Caucasoid | 99.99% | 9.0 | 7.0 | 88.07% | 3.76 | 2.51 | 100.0% | 12.77 | 9.96 |
| | | | | | | | | | |
| Taiwan | 97.77% | 6.22 | 4.02 | 67.88% | 2.51 | 0.93 | 99.29% | 8.73 | 6.09 |
| | | | | | | | | | |
| Taiwan Oriental | 97.77% | 6.22 | 4.02 | 67.88% | 2.51 | 0.93 | 99.29% | 8.73 | 6.09 |
| | | | | | | | | | |
| Thailand | 91.21% | 4.94 | 3.1 | 63.9% | 2.31 | 0.83 | 96.83% | 7.26 | 4.17 |
| | | | | | | | | | |
| Thailand Oriental | 91.21% | 4.94 | 3.1 | 63.9% | 2.31 | 0.83 | 96.83% | 7.26 | 4.17 |
| | | | | | | | | | |
| Tokelau | 0.0% | 0.0 | 0.0 | 55.11% | 1.82 | 0.67 | 55.11% | 1.82 | 0.67 |
| | | | | | | | | | |

| | | | | | | | | | |
|---|---|---|---|---|---|---|---|---|---|
| Tokelau Polynesian | 0.0% | 0.0 | 0.0 | 55.11 % | 1.82 | 0.6 7 | 55.11 % | 1.82 | 0.6 7 |
| | | | | | | | | | |
| Tonga | 0.0% | 0.0 | 0.0 | 71.91 % | 2.64 | 1.0 7 | 71.91 % | 2.64 | 1.0 7 |
| | | | | | | | | | |
| Tonga Polynesian | 0.0% | 0.0 | 0.0 | 71.91 % | 2.64 | 1.0 7 | 71.91 % | 2.64 | 1.0 7 |
| | | | | | | | | | |
| Tunisia | 97.52 % | 6.07 | 3.9 6 | 74.26 % | 2.83 | 1.1 7 | 99.36 % | 8.9 | 6.2 3 |
| | | | | | | | | | |
| Tunisia Arab | 97.52 % | 6.07 | 3.9 6 | 74.97 % | 2.86 | 1.2 | 99.38 % | 8.93 | 6.2 6 |
| | | | | | | | | | |
| Tunisia Berber | 0.0% | 0.0 | 0.0 | 74.47 % | 2.83 | 1.1 7 | 74.47 % | 2.83 | 1.1 7 |
| | | | | | | | | | |
| Turkey | 44.8% | 1.34 | 0.5 4 | 76.19 % | 2.96 | 1.2 6 | 86.85 % | 4.3 | 2.2 8 |
| | | | | | | | | | |
| Turkey Caucasoid | 44.8% | 1.34 | 0.5 4 | 76.19 % | 2.96 | 1.2 6 | 86.85 % | 4.3 | 2.2 8 |
| | | | | | | | | | |
| Uganda | 94.87 % | 5.9 | 3.5 6 | 0.0% | 0.0 | 0.0 | 94.87 % | 5.9 | 3.5 6 |
| | | | | | | | | | |
| Uganda Black | 94.87 % | 5.9 | 3.5 6 | 0.0% | 0.0 | 0.0 | 94.87 % | 5.9 | 3.5 6 |
| | | | | | | | | | |
| Ukraine | 0.0% | 0.0 | 0.0 | 50.64 % | 1.64 | 0.6 1 | 50.64 % | 1.64 | 0.6 1 |
| | | | | | | | | | |
| Ukraine Caucasoid | 0.0% | 0.0 | 0.0 | 50.64 % | 1.64 | 0.6 1 | 50.64 % | 1.64 | 0.6 1 |
| | | | | | | | | | |
| United Arab Emirates | 3.37% | 0.1 | 0.3 1 | 32.92 % | 0.99 | 0.4 5 | 35.19 % | 1.09 | 0.4 6 |
| | | | | | | | | | |
| United Arab Emirates Arab | 3.37% | 0.1 | 0.3 1 | 32.92 % | 0.99 | 0.4 5 | 35.19 % | 1.09 | 0.4 6 |
| | | | | | | | | | |

| | | | | | | | | | |
|---|---|---|---|---|---|---|---|---|---|
| United States | 99.1% | 7.4 | 5.17 | 88.1% | 3.76 | 2.52 | 99.89% | 11.16 | 8.14 |
| | | | | | | | | | |
| United States Amerindian | 99.67% | 6.85 | 4.82 | 42.79% | 1.43 | 0.52 | 99.81% | 8.28 | 6.05 |
| | | | | | | | | | |
| United States Asian | 97.46% | 6.47 | 4.07 | 78.84% | 3.13 | 1.42 | 99.46% | 9.6 | 6.54 |
| | | | | | | | | | |
| United States Austronesian | 0.0% | 0.0 | 0.0 | 58.09% | 2.06 | 0.72 | 58.09% | 2.06 | 0.72 |
| | | | | | | | | | |
| United States Black | 97.11% | 6.61 | 4.1 | 71.5% | 2.71 | 1.05 | 99.18% | 9.32 | 6.29 |
| | | | | | | | | | |
| United States Caucasoid | 99.83% | 8.57 | 6.53 | 90.15% | 3.91 | 3.01 | 99.98% | 12.48 | 9.63 |
| | | | | | | | | | |
| United States Hispanic | 98.92% | 7.01 | 4.78 | 72.95% | 2.79 | 1.11 | 99.71% | 9.79 | 6.79 |
| | | | | | | | | | |
| United States Mestizo | 99.23% | 7.1 | 5.01 | 72.23% | 2.75 | 1.08 | 99.79% | 9.85 | 6.88 |
| | | | | | | | | | |
| United States Polynesian | 99.57% | 7.39 | 5.42 | 73.18% | 2.75 | 1.12 | 99.88% | 10.14 | 7.15 |
| | | | | | | | | | |
| Venezuela | 90.04% | 3.97 | 3.0 | 3.01% | 0.09 | 0.31 | 90.34% | 4.06 | 3.02 |
| | | | | | | | | | |
| Venezuela Amerindian | 90.07% | 3.93 | 3.0 | 0.0% | 0.0 | 0.0 | 90.07% | 3.93 | 3.0 |
| | | | | | | | | | |
| Venezuela Caucasoid | 11.45% | 0.35 | 0.34 | 0.0% | 0.0 | 0.0 | 11.45% | 0.35 | 0.34 |
| | | | | | | | | | |

| | | | | | | | | | |
|---|---|---|---|---|---|---|---|---|---|
| Venezuela Mestizo | 9.75% | 0.29 | 0.33 | 0.0% | 0.0 | 0.0 | 9.75% | 0.29 | 0.33 |
| | | | | | | | | | |
| Venezuela Mixed | 0.0% | 0.0 | 0.0 | 3.17% | 0.1 | 0.31 | 3.17% | 0.1 | 0.31 |
| | | | | | | | | | |
| Vietnam | 91.82% | 5.0 | 3.15 | 54.44% | 1.9 | 0.66 | 96.27% | 6.89 | 3.94 |
| | | | | | | | | | |
| Vietnam Oriental | 91.82% | 5.0 | 3.15 | 54.44% | 1.9 | 0.66 | 96.27% | 6.89 | 3.94 |
| | | | | | | | | | |
| Wales | 1.0% | 0.03 | 0.3 | 0.0% | 0.0 | 0.0 | 1.0% | 0.03 | 0.3 |
| | | | | | | | | | |
| Wales Caucasoid | 1.0% | 0.03 | 0.3 | 0.0% | 0.0 | 0.0 | 1.0% | 0.03 | 0.3 |
| | | | | | | | | | |
| West Africa | 95.49% | 6.03 | 3.66 | 65.23% | 2.4 | 0.86 | 98.43% | 8.43 | 5.32 |
| | | | | | | | | | |
| West Indies | 98.98% | 7.5 | 5.25 | 69.22% | 2.59 | 0.97 | 99.69% | 10.09 | 6.94 |
| | | | | | | | | | |
| World | 98.55% | 6.9 | 4.54 | 81.81% | 3.32 | 1.65 | 99.74% | 10.22 | 7.07 |
| | | | | | | | | | |
| Zambia | 98.1% | 6.42 | 4.16 | 0.0% | 0.0 | 0.0 | 98.1% | 6.42 | 4.16 |
| | | | | | | | | | |
| Zambia Black | 98.1% | 6.42 | 4.16 | 0.0% | 0.0 | 0.0 | 98.1% | 6.42 | 4.16 |
| | | | | | | | | | |
| Zimbabwe | 93.79% | 5.43 | 3.37 | 68.3% | 2.51 | 0.95 | 98.03% | 7.95 | 4.84 |
| | | | | | | | | | |
| Zimbabwe Black | 93.79% | 5.43 | 3.37 | 68.3% | 2.51 | 0.95 | 98.03% | 7.95 | 4.84 |
| | | | | | | | | | |
| **Average** | **63.06** | **4.0** | **2.71** | **55.3** | **2.11** | **1.04** | **82.92** | **6.11** | **4.02** |
| **Standard deviation** | **43.68** | **3.13** | **2.27** | **30.26** | **1.26** | **0.83** | **25.25** | **3.59** | **2.85** |

**Table S6.** Docking score of vaccine with MHC-I.

| Cluster | Members | Representative | Weighted Score |
|---|---|---|---|
| **0** | 138 | Center | -733.6 |
| | | Lowest Energy | -799.3 |
| **1** | 95 | Center | -586.8 |
| | | Lowest Energy | -638.1 |
| **2** | 71 | Center | -608.9 |
| | | Lowest Energy | -608.9 |
| **3** | 59 | Center | -596.1 |
| | | Lowest Energy | -624.6 |
| **4** | 42 | Center | -556.0 |
| | | Lowest Energy | -642.2 |
| **5** | 39 | Center | -590.9 |
| | | Lowest Energy | -627.9 |
| **6** | 36 | Center | -650.3 |
| | | Lowest Energy | -658.9 |
| **7** | 35 | Center | -674.5 |
| | | Lowest Energy | -674.5 |
| **8** | 34 | Center | -665.4 |
| | | Lowest Energy | -665.4 |
| **9** | 31 | Center | -579.0 |
| | | Lowest Energy | -606.3 |
| **10** | 30 | Center | -699.5 |
| | | Lowest Energy | -699.5 |

**Table S7.** Docking score of vaccine with MHC-II.

| Cluster | Members | Representative | Weighted Score |
|---|---|---|---|
| **0** | 87 | Center | -696.0 |
| | | Lowest Energy | -821.4 |
| **1** | 76 | Center | -648.9 |

| Cluster | Members | Representative | Weighted Score |
|---|---|---|---|
| | | Lowest Energy | -789.4 |
| **2** | 68 | Center | -743.2 |
| | | Lowest Energy | -743.2 |
| **3** | 64 | Center | -796.4 |
| | | Lowest Energy | -796.4 |
| **4** | 53 | Center | -748.8 |
| | | Lowest Energy | -748.8 |
| **5** | 46 | Center | -755.9 |
| | | Lowest Energy | -794.5 |
| **6** | 40 | Center | -645.6 |
| | | Lowest Energy | -713.9 |
| **7** | 38 | Center | -786.6 |
| | | Lowest Energy | -786.6 |
| **8** | 37 | Center | -696.5 |
| | | Lowest Energy | -752.1 |
| **9** | 29 | Center | -650.5 |
| | | Lowest Energy | -789.0 |
| **10** | 29 | Center | -642.3 |
| | | Lowest Energy | -790.5 |

**Table S8.** Docking score of vaccine with TLR-4.

| Cluster | Members | Representative | Weighted Score |
|---|---|---|---|
| **0** | 82 | Center | -691.7 |
| | | Lowest Energy | -783.6 |
| **1** | 46 | Center | -758.5 |
| | | Lowest Energy | -758.5 |
| **2** | 44 | Center | -607.5 |

| Cluster | Members | Representative | Weighted Score |
|---|---|---|---|
| | | Lowest Energy | -803.3 |
| **3** | 36 | Center | -646.0 |
| | | Lowest Energy | -699.0 |
| **4** | 35 | Center | -637.6 |
| | | Lowest Energy | -704.1 |
| **5** | 34 | Center | -695.4 |
| | | Lowest Energy | -703.4 |
| **6** | 32 | Center | -678.9 |
| | | Lowest Energy | -752.2 |
| **7** | 31 | Center | -667.6 |
| | | Lowest Energy | -832.6 |
| **8** | 31 | Center | -704.5 |
| | | Lowest Energy | -711.0 |
| **9** | 30 | Center | -709.4 |
| | | Lowest Energy | -709.4 |
| **10** | 30 | Center | -691.2 |
| | | Lowest Energy | -691.2 |
